# Quantitative analysis of heme and hemoglobin for the detection of intravascular hemolysis

**DOI:** 10.1016/j.aca.2024.342766

**Published:** 2024-05-21

**Authors:** Marie-T. Hopp, Sonali M. Vaidya, Karina M. Grimmig, Lasse J. Strudthoff, Johanna C. Clauser, Xiaojing Yuan, Sneha Singh, Jens Müller, Johannes Oldenburg, Iqbal Hamza, Diana Imhof

**Affiliations:** a Pharmaceutical Biochemistry and Bioanalytics, Pharmaceutical Institute, University of Bonn, D-53121, Bonn, Germany; b Department of Chemistry, Institute for Integrated Natural Sciences, University of Koblenz, D-56070, Koblenz, Germany; c Department of Cardiovascular Engineering, Institute of Applied Medical Engineering, Medical Faculty RWTH Aachen University, D-52074, Aachen, Germany; d Center for Blood Oxygen Transport and Hemostasis, Department of Pediatrics, University of Maryland School of Medicine, US-21201, Baltimore, MD, USA; e Institute of Experimental Hematology and Transfusion Medicine, University Hospital Bonn, D-53127, Bonn, Germany

**Keywords:** Diagnosis, Heme, Hemoglobin, Hemolysis, Quantification methods

## Abstract

**Background:**

Intravascular hemolysis is associated with massive release of hemoglobin and consequently labile heme into the blood, resulting in prothrombotic and proinflammatory events in patients. Though heme is well-known to participate in these adverse effects, it is not monitored. Instead, haptoglobin and hemoglobin serve as clinical biomarkers. The quantification of labile heme together with hemoglobin, however, should be considered in clinical diagnosis as well, to obtain a complete picture of the hemolytic state in patients. So far, quantification techniques for labile heme were not yet systematically analyzed and compared for their clinical application potential, especially in the presence of hemoglobin.

**Results:**

Two commercial assays (Heme Assay Kit^®^, Hemin Assay Kit^®^) and five common approaches (pyridine hemochromogen assay, apo-horseradish peroxidase-based assay, UV/Vis spectroscopy, HPLC, mass spectrometry) were analyzed concerning their linearity, accuracy, and precision, as well as their ability to distinguish between hemoglobin-bound heme and labile heme. Further, techniques for the quantification of hemoglobin (Harboe method, SLS method, Hemastix^®^) were included to study their selectivity for hemoglobin and potential interference by the presence of labile heme. Both, indirect and direct approaches were suitable for the determination of a wide concentration of heme (~0.02–45 μM) and hemoglobin (~0.002–17 μM). A clear distinction between hemoglobin-bound heme and labile heme with one method was not possible. Thus, a novel combined approach is presented and applied to human and porcine plasma samples for the determination of hemoglobin and labile heme.

**Significance:**

Our results demonstrate the need to develop improved techniques to differentiate labile and protein-bound heme for early detection of intravascular hemolysis. Here, we present a novel strategy by combining two spectroscopic methods, which is most reliable as an easy-to-use tool for the determination of hemoglobin and heme levels in plasma samples for the diagnosis of intravascular hemolysis and in basic biomedical research.

## Graphical Abstract

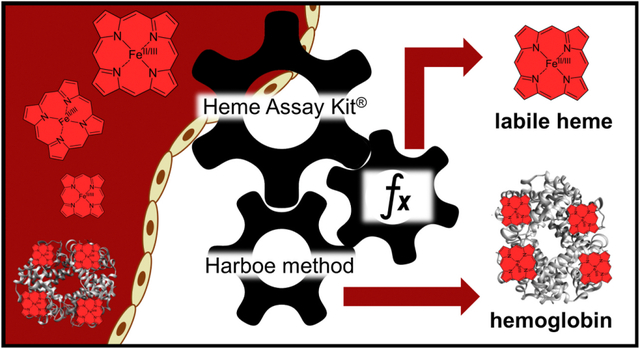

## 1. Introduction

Heme plays a vital role in numerous physiological processes, such as oxygen transport, when present in hemoglobin [1]. Hemoglobin, in turn, resides in the prodigious amount of ~300 million molecules per red blood cell (RBC) [2]. The blood hemoglobin content is a crucial parameter for hematological diagnostics in hemolytic disorders such as anemias and hemolytic reactions following RBC transfusions [3]. Hemolysis is caused by premature RBC lysis, causing massive hemoglobin and labile heme accumulation in the blood [4,5]. Under these conditions, endogenous defense mechanisms become exhausted, and the plasma heme concentration may reach up to 170 μM [6,7]. Extracellular hemoglobin and heme can trigger prooxidant, proinflammatory, and procoagulant processes that are associated with severe complications [8]. Thus, it is of utmost importance to implement a point-of-care method to determine emerging heme levels with the concomitant presence of hemoglobin. However, the presence of heme in hemoglobin poses additional difficulties [9]. While commonly used direct methods refer to immediate analyte determination (e.g., UV/Vis spectroscopy, mass spectrometry (MS), high-performance liquid chromatography (HPLC)), indirect methods use a chemical conversion and/or complex formation of the analyte with assay reagents. Examples include the pyridine hemochromogen assay [10–12], Hemastix^®^ test strips [13], Hemin Assay Kit^®^ and apo-horseradish peroxidase (apoHRP)-based assay [14], sodium lauryl sulphate (SLS) method [15], and Heme Assay Kit^®^ [9].

In this study, we present the first in-depth investigation and analytical comparison of the hemoglobin and heme determination methods in standard and plasma samples. To our knowledge, this is the first in-depth investigation and comparison of the methods available to determine hemoglobin and heme concentrations in standard and plasma samples. Our studies reveal that the combination of the Harboe method [16] for the detection of hemoglobin in combination with the Heme Assay Kit^®^ and the application of an adapted calculation scheme for the estimation of labile heme provides the quantification of both molecules in a more accurate way than the application of either method alone. This strategy is thus recommended for conditions characterized by high levels of intravascular extracellular heme with the concomitant presence of high hemoglobin levels in a highly complex biological matrix.

## 2. Materials and methods

### 2.1. Reagents and general procedures

Details are provided in the Supplementary material.

### 2.2. Plasma samples

Porcine plasma samples were taken from German Landrace pigs (Supplementary methods). Human citrated plasma samples were provided by the Institute of Experimental Hematology and Transfusion Medicine, University Hospital Bonn. All procedures were conducted in accordance with the Helsinki Declaration.

### 2.3. UV/Vis spectroscopy

For quantification, 200 μL of heme (10–40 μM) and hemoglobin (1–7 μM) in PBS, pH 7.4, as well as mixtures of heme (5 μM, 10 μM, 15 μM) and hemoglobin (2 μM, 5 μM, 10 μM) were applied on a 96-well microtiter plate (Greiner Bio-One GmbH, Frickenhausen, Germany). According to the observed maxima, the absorbance was measured at 380 nm and 405 nm. The standard equations were used for analyte content calculation from mixtures. A 100 μL set-up was established as well (Fig. S1).

### 2.4. Harboe method

Following the conventional protocol [16], hemoglobin (1.3–16.5 μM) as well as mixtures of hemoglobin (2 μM, 5 μM, 10 μM) and heme (5 μM, 10 μM, 15 μM) were analyzed with slight modifications. In brief, 100 μL of each sample or mixture was applied on a microtiter plate and the absorbance (380, 415, 450 nm) was measured. Hemoglobin levels were calculated using the equation: c(Hb) [μM] = 0.155 × (83.6 × (2 × *A*_*415*_ – *A*_*380*_ – *A*_*450*_)) [17]. Measurements were also performed in water with 0.1 % Na_2_CO_3_ (as Harboe [16]), in PBS with 0.1 % Na_2_CO_3_, and in Tris-HCl buffer (288 μM, pH 7.4) (Fig. S2).

### 2.5. HPLC and electrospray ionization (ESI)-MS

Heme (20–45 μM) and hemoglobin (0.5–6 μM) standard solutions (400 μL) as well as mixtures of heme (5 μM, 10 μM, 15 μM) and hemoglobin (2 μM, 5 μM) were injected into a HPLC (LC-20A, Shimadzu, Duisburg, Germany) equipped with a C4 column (NUCLEODUR^®^ 300–5 C4 ec, 250 × 4.6 mm, 300 Å pore size, 5 μm particle size). Eluents used for HPLC were 0.1 % trifluoroacetic acid (TFA) in water (A) and 0.1 % TFA in acetonitrile (B). A gradient system of 30–70 % eluent B in 40 min with a flow rate of 1 mL min^−1^ was applied. The area under the curve (AUC) at 220 nm was analyzed with LabSolutions (Shimadzu). For ESI-MS analysis, a protocol adapted from Fyrestam et al. [18] was used (Supplementary material, Table S1).

### 2.6. Pyridine hemochromogen assay

Following an established protocol [12,19], heme (7.5–37.5 μM) and hemoglobin (1.3–12.7 μM) standards were mixed 1:1 with a solution, consisting of NaOH (0.2 M), pyridine (40 %) and potassium ferricyanide (500 μM), in a cuvette and a spectrum (500–600 nm) of this oxidized sample was recorded. Afterwards, the sample was mixed with 10 μL sodium dithionite (0.5 M in 0.5 M NaOH) solution. Spectra (500–600 nm) of these reduced samples were measured in 1-min-intervals for 5 min. The same was applied for heme (10 μM, 15 μM) and hemoglobin (2 μM, 5 μM, 10 μM) mixtures.

### 2.7. Peroxidase activity-based approaches

Hemastix^®^ reagent strips were dipped into hemoglobin (0.25 nM-2.5 μM) and heme (1 nM-10 μM) solutions. In addition, mixtures of both (heme + hemoglobin: 50 nM + 50 nM, 50 nM + 25 nM, and 5 μM + 6.25 nM) were tested. Following the manufacturer’s instructions, a color change of the test strip from yellow to green/blue was considered as “positive” for hemoglobin [13]. Following an available protocol [14,20, 21], the apoHRP-based assay was performed with heme and hemoglobin as well as mixtures thereof using 3,3′,5,5′-tetramethylbenzidine (TMB) and *o*-dianisidine dihydrochloride as substrates (Supplementary methods). In addition, the Hemin Assay Kit^®^ was performed (Supplementary methods).

### 2.8. Surfactant-based assays

For the SLS method, hemoglobin (10 μL) was transferred into the reagent (2.25 mL of 2.08 mM SLS and 0.075 % tergitol in 30 mM phosphate buffer, pH 7.3), as provided in round cuvettes (Diaglobal HB kit). The method was slightly modified to establish a standard curve for hemoglobin (0.5–6 μM) and heme (10–45 μM). In brief, the analyte and the reagent (100 μL each) were mixed and a spectrum (300–600 nm) was recorded. The absorbance at 395 nm (heme) and 413 nm (hemoglobin) was considered. The impact of heme on the hemoglobin determination was analyzed by adding heme (10 μM, 15 μM, 20 μM) to hemoglobin (2 μM, 4 μM). Furthermore, the commercial Heme Assay Kit^®^ was performed (Supplementary methods) and its ingredients were analyzed by ESI-MS (Fig. S3).

### 2.9. Determination of hemoglobin and heme levels in plasma samples

22 porcine (**P1**–**P22**) and 20 human (**H1**–**H20**) plasma samples were analyzed for their hemoglobin and labile heme content by the combination of the Harboe method [16,17] and the Heme Assay Kit^®^. If necessary, samples were diluted with PBS. As a control, hemoglobin and labile heme levels were also determined in three non-hemolytic (**H21**–**H23**) and three hemolytic (**H24**–**H26**) human plasma samples upon mixture with heme solutions of defined concentration (2 μM, 5 μM, 10 μM, 15 μM).

### 2.10. Statistical analysis

Results are shown as mean ± standard deviation (SD) from triplicates. Significance was determined by ordinary one-way variance analysis (ANOVA) with Tukey’s multiple comparisons test using GraphPad prism (Version 9.3.1) [22]. Statistical significance levels were represented as *p < 0.05, **p < 0.01, ***p < 0.001, and ****p < 0.0001.

## 3. Results

**Fig. 1.**
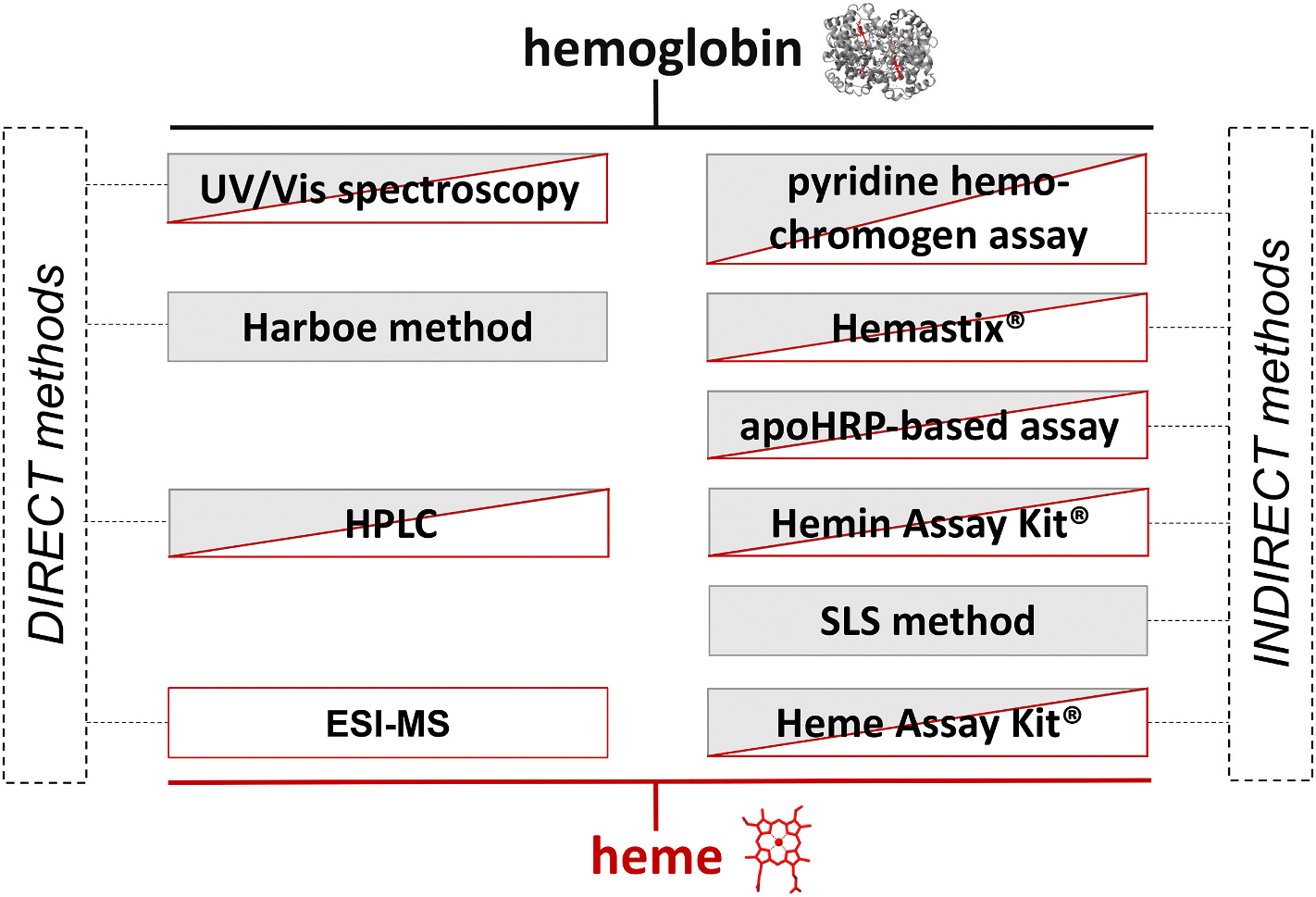
Overview of heme and hemoglobin quantification methods. Commonly used direct and indirect methods for the quantification of heme (red) and hemoglobin (grey) were compared in this study. It was highlighted that most of these techniques detect non-specifically heme and hemoglobin but without the ability to distinguish between both analytes.

To evaluate a set of heme and hemoglobin quantification methods (Fig. 1, Table S2), the ICH guidelines [23,24] were considered.

### 3.1. Hemoglobin and heme quantification by direct methods

**Fig. 2.**
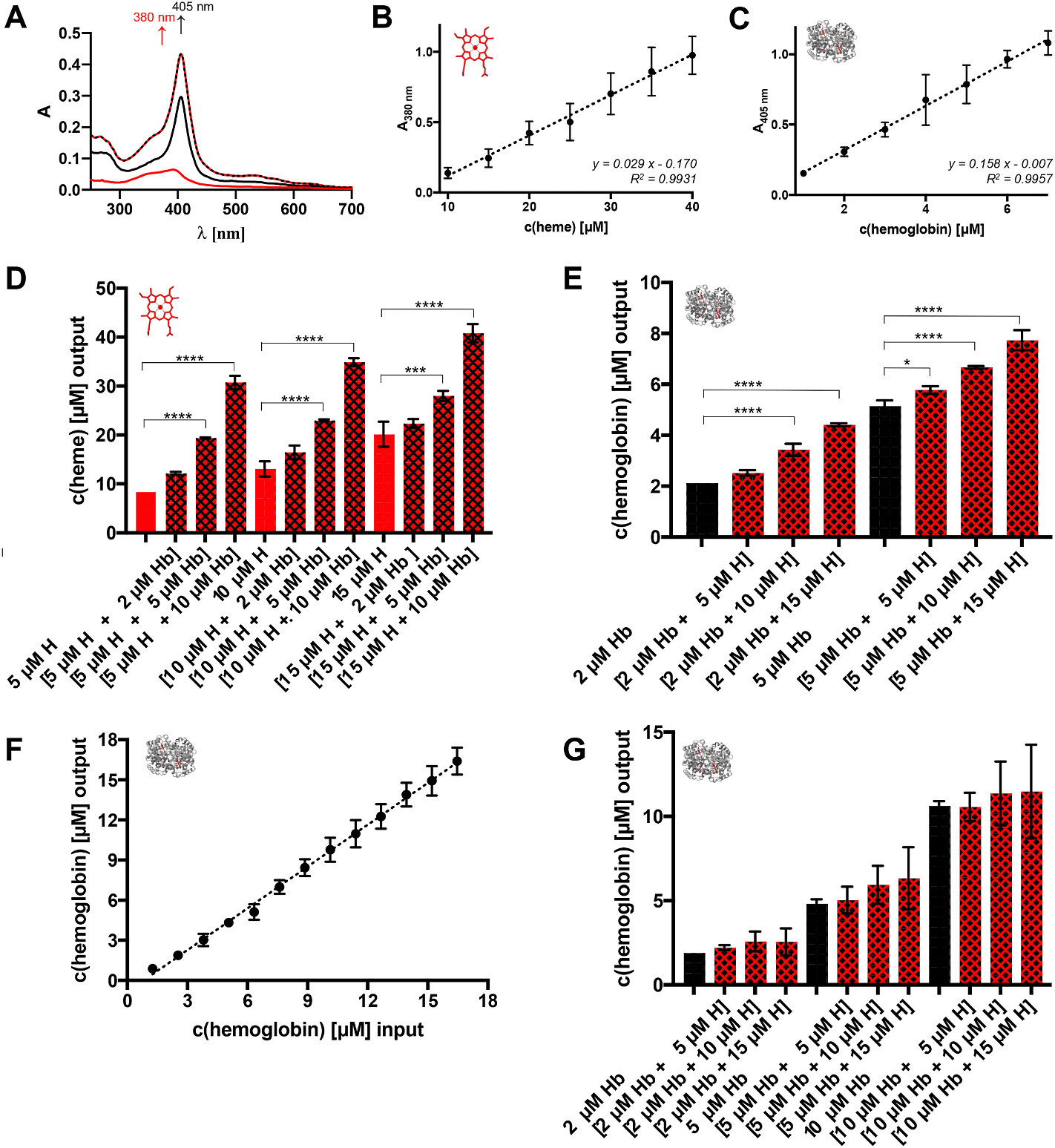
UV/Vis spectroscopic quantification of heme and hemoglobin. (A) The characteristic spectra of heme and hemoglobin enable the detection of both analytes. Heme (red) has the typical broad Soret band at ~380 nm, whereas the absorbance maximum of hemoglobin (black) is at ~405 nm in PBS. Through transient interaction, a mixture of hemoglobin with heme (dashed line) results in a hyperchromic shift of the absorbance maximum at ~405 nm. (B) Calibration curve (*y* = 0.029 *x* - 0.170) for the spectroscopic detection of heme at 380 nm. It shows linearity in the range of 10–40 μM heme in the 200 μL approach. (C) Calibration curve (*y* = 0.158 *x* - 0.007) for the spectroscopic detection of hemoglobin at 405 nm. It shows linearity in the range of 1–7 μM hemoglobin in the 200 μL approach. (D) Heme determination from hemoglobin-heme mixtures by measuring the absorbance at 380 nm. The addition of hemoglobin (2–10 μM) to heme (5–15 μM) solutions significantly influences the amount of heme being detected. (E) The same is observed when adding heme (5–15 μM) to hemoglobin (2–5 μM), analyzed by the absorbance at 405 nm. (F) According to Lambert-Beer’s law, hemoglobin solutions (in PBS) can be quantified with the Harboe method within the range of ~1.27–16.5 μM. (G) The addition of heme (5–15 μM) to hemoglobin (2–10 μM) does not significantly influence the hemoglobin concentration determined by the Harboe method. H, heme; Hb, hemoglobin; *p < 0.05, ***p < 0.001, and ****p < 0.0001.

Heme and hemoglobin showed absorbance maxima at 380 nm and 405 nm, respectively, in PBS. The incubation of hemoglobin with heme resulted in a hyperchromic shift (Fig. 2A) [25]. Thus, heme could be quantified in the range of 10–40 μM (Fig. 2B) at 380 nm with a mean recovery rate (MRR) of 100.43 ± 15.35 %, while the hemoglobin absorbance was linear in the range of 1–7 μM (Fig. 2C) at 405 nm with a MRR of 101.31 ± 12.81 % (Table S3).

In both cases, the MRR showed <15 % deviation proving linearity [23,24]. Using hemoglobin-heme mixtures, an additive effect was evident (Fig. 2D and E). However, this increase was neither reflecting the amount added, nor was it proportionally increasing. Accordingly, added heme interfered with the analyte determination. Using a 100 μL set-up, the quantification ranges shifted to 50–90 μM for heme and 1.25–10 μM for hemoglobin (Fig. S1, Table S4). Microtiter plates from different manufacturers are characterized by different pathlengths, which was considered while evaluating the measurements. The mixture of both analytes did not result in any significant effect (Fig. 2D).

Using the Harboe method [16,17] in PBS (Fig. S4), hemoglobin was detected in a range of 1.3–16.5 μM (Fig. 2F) with a MRR of 89.54 ± 7.31 %, representing an extended range than demonstrated earlier [16]. In the lower range (1.3–6.3 μM), however, the deviation was beyond 15 %, which made it less reliable in this range (Table S5). Addition of heme to hemoglobin revealed an increase of standard deviations (Fig. 2G). With other buffers, a slightly increased range of 2.5–17.5 μM for hemoglobin was detected (Fig. S2). Yet, while Tris-HCl led to an overestimation, the use of PBS with Na_2_CO_3_ and pure Na_2_CO_3_ solution resulted in an underestimation of hemoglobin.

**Fig. 3.**
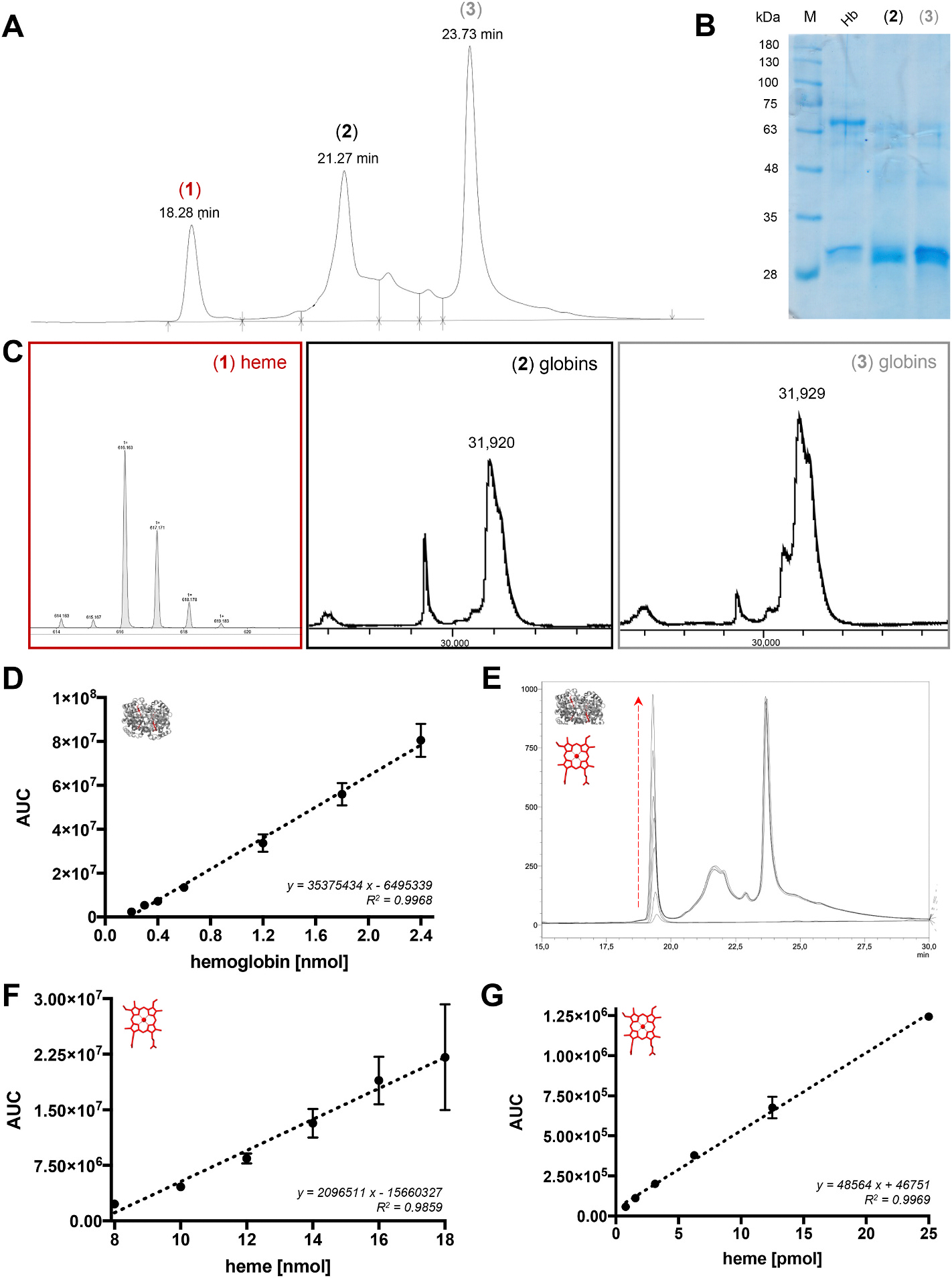
HPLC- and ESI-MS-assisted heme and hemoglobin quantification. (A) The HPLC chromatogram of commercially obtained hemoglobin is characterized by three main peaks (1)–(3). The elution time within the applied gradient system of 30–70 % acetonitrile (+0.1 % TFA) in water (+0.1 % TFA) is depicted for each peak. (B) The SDS-PAGE gel of hemoglobin in comparison to peak (2) and (3) from HPLC separation shows that commercial hemoglobin mainly contains the tetrameric (~64 kDa, black box) but also the dimeric (~32 kDa, black arrow) form, whereas the HPLC-separated fractions (2) and (3) predominantly contain the dimeric form of hemoglobin. (C) The MALDI-TOF-MS analysis reveals that heme occurs separately from the globin components of hemoglobin, since only the peak (1) shows an intense heme signal with an *m/z* of 616.16, while the fractions (2) and (3) contain the protein moieties. Zoom-Ins into the dimeric mass signal are depicted, the complete spectra are found in Supplementary Fig. 6 (D). The calibration curve of hemoglobin for HPLC analysis (*y* = 35375434 *x* - 6495339) is valid in the range of 0.2–2.4 pmol hemoglobin at 220 nm using the above-mentioned gradient system and a C4 column. (E) The addition of heme (5–15 μM) to hemoglobin (5 μM) can be observed by the increase of peak (1). However, the differentiation between hemoglobin-derived heme and labile heme is not possible. (F) Under the same conditions, the heme calibration curve (*y* = 2096511 *x* - 15660327) is applicable for the range of 8–18 pmol heme. (G) With ESI-MS analysis, heme standard solutions (in 50 % acetonitrile/water) can be quantified in the range of 0.8–25 pmol (*y* = 48564 *x* + 46751). Hb, hemoglobin; M, marker.

The use of hemoglobin without prior purification (Fig. 3A and B; Fig. S5), as commonly used [26,27], is critical. Although the hemoglobin tetramer appears as the major component of the commercial hemoglobin, the dimeric form was present (Fig. 3B and C), which was the predominant form after HPLC separation (Fig. 3B, Fig. S6). In addition to the hemoglobin dimers, heme eluted separately from the globins (Fig. 3A–C; Fig. S6) [18]. Although hemoglobin (0.5–6 μM, ≙ ~0.2–2.4 nmol) could be determined by HPLC (Fig. 3D; Fig. S7) with a MRR of 103.32 ± 9.83 %, the instability of hemoglobin prevented unequivocal differentiation from simultaneously occurring heme together with other degradation products (e.g., hemoglobin dimers and monomers; Fig. 3A–C). As such, heme addition to hemoglobin was observed as an increased peak but could not be distinguished from hemoglobin-derived heme (Fig. 3E). The concentration of heme concerning quantification limits varied from 20 μM to 45 μM for HPLC (≙ ~8–18 nmol; Fig. 3F; Fig. S7) with a MRR of 101.97 ± 7.08 % (Table S6) and from 39 nM to 1250 nM for ESI-MS (≙ ~0.8–25 pmol; Fig. 3G; Table S7), with the latter revealing a calculated LOD of ~0.96 pmol. Recalculation of the heme amount in hemoglobin with the HPLC heme calibration curve, revealed six heme molecules per hemoglobin molecule instead of the expected four (Table S8).

### 3.2. Hemoglobin and heme quantification by indirect methods

**Fig. 4.**
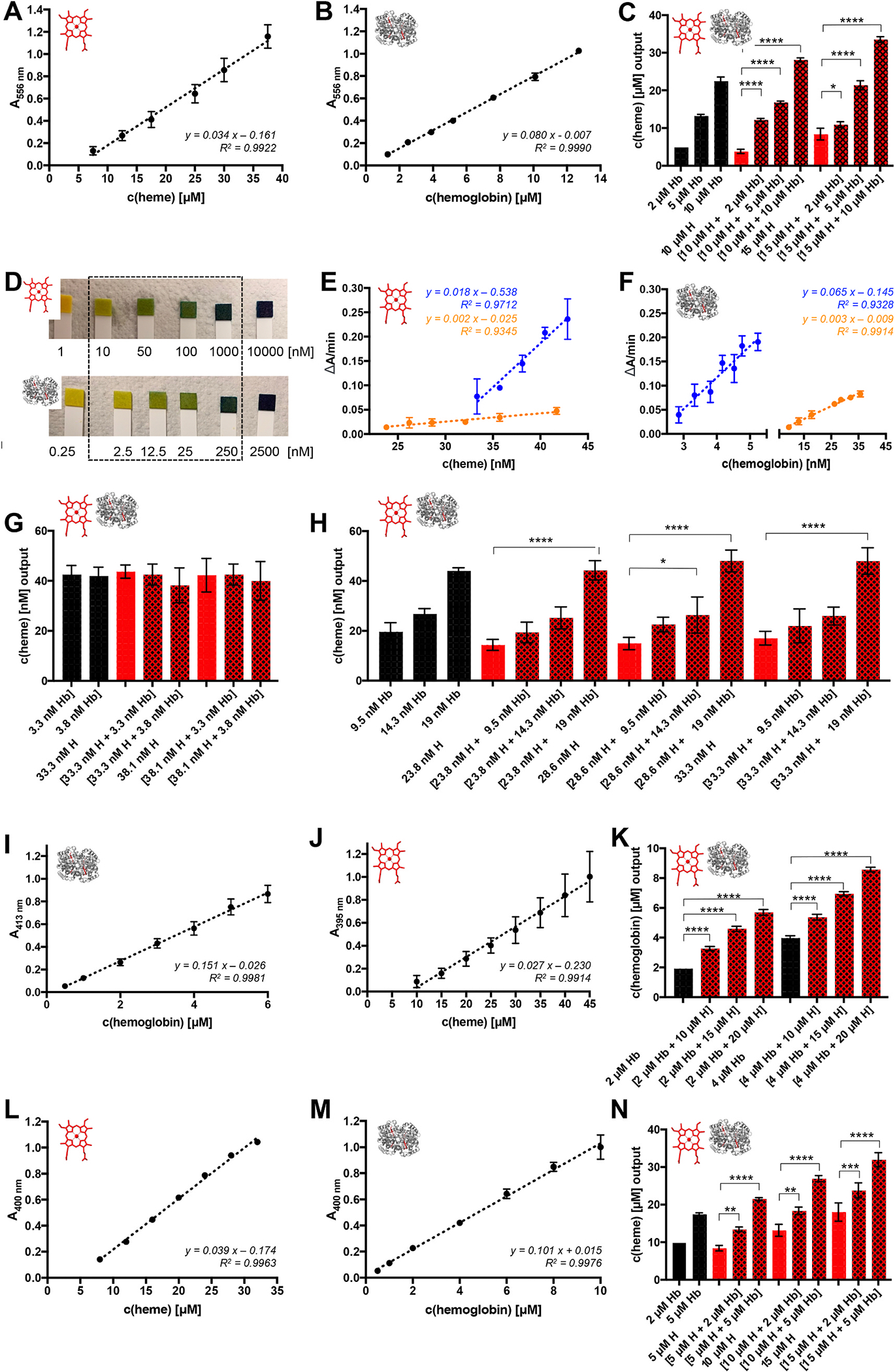
Heme and hemoglobin quantification by indirect methods. With the pyridine hemochromogen assay, heme can be quantified in the range of 7.5–37.5 μM (A) and hemoglobin in the range of 1.3–12.7 μM (B). Depicted is the evaluation at the absorbance maximum of the reduced sample at 556 nm. Other commonly used evaluation techniques are displayed in Supplementary Fig. 8 (C). Mixtures of heme (10–15 μM) and hemoglobin (2–10 μM) show the additive effect of both components. Data are evaluated using the linear heme calibration equation (*y* = 0.034 *x* - 0.161). (D) With the Hemastix^®^ test strips, both components are detected by a green to blue color change in the range of 10–1000 nM (heme) and 2.5–250 nM (hemoglobin) through their peroxidase-like activity. Examples for the analysis of mixtures are found in Supplementary Fig. 9 (E). With the apoHRP-based assay, heme was detected in the range of ~33–43 nM by using TMB as the substrate (blue) and ~24–42 nM by using *o*-dianisidine as the substrate (orange). (F) For hemoglobin, also a concentration-dependent effect could be observed in the range of ~3–5 nM by using TMB as the substrate (blue) and ~9.5–36 nM by using *o*-dianisidine as the substrate (orange). (G) Combining 1:10 mixtures of hemoglobin and heme with the apoHRP-based assay using TMB as the substrate did not show any additive effect. (H) In contrast, using analyte combinations in the concentration range of the *o*-dianisidine-based assay, hemoglobin addition increased the determined heme concentration. However, the heme detection level is the same in the heme-hemoglobin combinations and the respective pure hemoglobin solutions. (I) With the modified, hemoglobin detection SLS method, the absorbance at 413 nm was used to quantify hemoglobin in standard solutions, which worked for 0.5–6 μM hemoglobin. (J) With the modified SLS method, the absorbance at 395 nm was used to quantify heme in standard solutions (10–45 μM). (K) Heme (10–20 μM) significantly increased the hemoglobin (2–4 μM) result from hemoglobin-heme mixtures. (L) With the commercially available Heme Assay Kit^®^ heme could be quantified at 400 nm within the range of 8–32 μM. (M) Hemoglobin could be quantified with the Heme Assay Kit^®^ at 400 nm within the range of 0.5–10 μM. (N) Using the Heme Assay Kit^®^ for hemoglobin-heme mixtures revealed again a significant additive effect when determining the heme concentration from the mixtures. H, heme; Hb, hemoglobin; *p < 0.05, **p < 0.01, ***p < 0.001, and ****p < 0.0001.

With the pyridine hemochromogen assay [10–12], both analytes could be quantified in the range of 7.5–37.5 μM (heme) and 1.3–12.7 μM (hemoglobin) (Fig. 4A and B).

Using *ε*_556_ = 34.1 mM^−1^ cm^−1^ (heme) and *ε*_556_ = 80.4 mM^−1^ cm^−1^ (hemoglobin), the MRRs were 72.43 ± 12.66 % and 98.30 ± 5.20 % (Table S9), whereas the application of the linear equations resulted in better recovery rates with 101.89 ± 12.69 % and 95.90 ± 5.20 % (Table S10), respectively. Similar results were obtained by using the absorbance difference between the reduced and oxidized state (Fig. S8, Table S11). Conversely, application of the earlier published [12,28] *ε*_557_ = 34.7 mM^−1^ cm^−1^ resulted in a MRR of only 69.72 ± 12.19 % (Table S12), while the use of the published [12,29] *ε*_557–540_ = 23.98 mM^−1^ cm^−1^ revealed an acceptable recovery (99.68 ± 11.61 %) in the range of 25–37.5 μM heme (Table S13; Fig. S8). The heme concentration determined in hemoglobin-heme mixtures was significantly higher compared to the individual components (Fig. 4C). On the other hand, heme determination from hemoglobin solutions revealed a ratio of ~1: 2.2 (hemoglobin:heme) compared to the expected 1:4 ratio (Fig. 4C; Figs. S8D and E), which has been described for bovine hemoglobin as well [19].

The Hemastix^®^ reagent strips, developed for hemoglobin detection, showed positive results for hemoglobin (2.5–250 nM) and heme (10–1000 nM) as well as combinations thereof (Fig. 4D, Fig. S9). Essentially, 2.5 μM hemoglobin and 10 μM heme (each alone) displayed the same dark blue color on the test strip as 0.25 μM hemoglobin and 1.0 μM heme solutions, highlighting the upper quantification limit for heme at 1 μM and for hemoglobin at 250 nM. In contrast to the test strips, the apoHRP-based assay detects heme indirectly by the activity of reconstituted HRP (Fig. 4 E, F; Fig. S10). The heme quantification range with *o*-dianisidine (~23.8–41.7 nM) was slightly broader than with TMB (~33.3–42.9 nM; Fig. 4E). The MRR for the assay with TMB (100.65 ± 3.26 %) was within an acceptable range, whereas the assay with *o*-diansidine revealed a MRR of 84.20 ± 11.00 % and thus did not meet the ICH requirements [23,24]. As suggested earlier [14], a substrate conversion was also observed in the presence of hemoglobin in the range of ~2.9–5.2 nM (TMB) and ~9.5–35.7 nM (*o*-dianisidine) (Fig. 4F). The MRRs for both approaches were acceptable (Table S14, Table S15). Analysis of hemoglobin-heme mixtures revealed no additive effect. In contrast, it seems that the activity of HRP reconstituted by hemoglobin-derived heme was determined from the mixtures (Fig. 4G and H). If this activity differed from heme, it resulted in an increase of the detected heme concentration (Fig. 4H). A correlation between the activity of HRP reconstituted by hemoglobin-derived heme versus HRP reconstituted by labile heme could not be observed in the TMB-based assay, since the recalculation of heme from hemoglobin revealed much too high heme concentrations (3.629 × *c(Hb)* + 21.86 nM; Fig. S10). Another variant of the apoHRP-based assay, in which the provided kit enzyme mix contains only apoHRP, as was identified herein by a combination of MALDI-TOF-MS and SDS-PAGE (Fig. S10), is the commercial Hemin Assay Kit^®^ (Fig. S11). During pipetting, however, the formation of bubbles from PEG or PEG-related detergents in the kit buffer (Fig. S12) impaired the results making it much less reliable (Figs. S10C and D; Table S16).

The SLS method is a commonly used approach for hemoglobin quantification, which detects high hemoglobin levels (1.86–15.5 mM according to the manufacturer) at 546 nm (Fig. S13), which makes it applicable for total hemoglobin determination but not for extracellular hemoglobin under hemolytic conditions. Thus, the method was modified towards the observed Soret band shifts to ~413 nm (hemoglobin) and ~395 nm (heme), which provided more reliable results in the range of 0.5–6 μM (hemoglobin, MRR 100.23 ± 10.46 %) and 10–45 μM (heme, MRR 99.41 ± 14.48 %) (Fig. 4I and J; Table S17). However, due to the absorbance maxima proximity, the mixture with heme resulted in an overestimation of hemoglobin (Fig. 4K). We identified the Heme Assay Kit^®^ herein as a surfactant-based heme detection kit which uses an approach with Triton X-100 in alkaline solution (Fig. S3) that has been described in 1999 [30]. In contrast to the detection range given by the manufacturer (0.6–125 μM), we only quantified heme in the range of 8–32 μM (MRR 100.46 ± 3.56 %) following the validity limits of Lambert-Beer’s law (Fig. 4L; Table S18). The same approach was applied to hemoglobin, which could be quantified from 0.5 μM to 10 μM with a MRR of 98.00 ± 11.58 % (Fig. 4M). Since both analytes were detected at 400 nm, a significant absorbance increase could be observed with mixtures (Fig. 4N; Fig. S14A), impeding precise quantification of the individual analytes. The detected heme amount in hemoglobin using the Heme Assay Kit^®^ was <4:1 heme:hemoglobin (Fig. S14B) and thus, not able to precisely determine total heme from mixtures.

Since the Harboe method served as a reliable approach for hemoglobin determination with acceptable recovery rates even in the presence of heme, the combination of the Harboe method and the Heme Assay Kit^®^ as well as its application towards hemoglobin-heme mixtures was tested. Hemoglobin was determined by the Harboe method, while for labile heme calculation with the Heme Assay Kit^®^ an established equation was used, which was based on the respective linear regression curves (Fig. 4L, M; Eq. 1).

$$c\;\left(\text{labile}\;\text{heme}\right)\;[\mu M]={DF}_{\left(HAK\right)}\times \left(25.64\times A_{400\;nm}-2.59\times \left(c_{\left(Hb,HAR-\text{BOE}\right)}\right.\right.\left.\left./{DF}_{\left(HAK\right)}\right)+4.85\right)$$

**Eq. 1:** Equation for the use of the Heme Assay Kit^®^ for labile heme quantification combined with the equation from the Harboe method for the hemoglobin determination. A, absorbance; DF, dilution factor; HAK, Heme Assay Kit^®^; Hb, hemoglobin.

With Eq. 1, labile heme could be assessed from hemoglobin-heme mixtures with a MRR of 93.35 ± 25.81 % in PBS-based solutions (Table S19) and a MRR of 103.04 ± 9.71 % in plasma samples spiked with defined amounts of heme (Table S20), which is superior to any other method and thus was subsequently used to quantify hemoglobin and labile heme in plasma samples.

### 3.3. Quantification of hemoglobin and labile heme in plasma samples

**Fig. 5.**
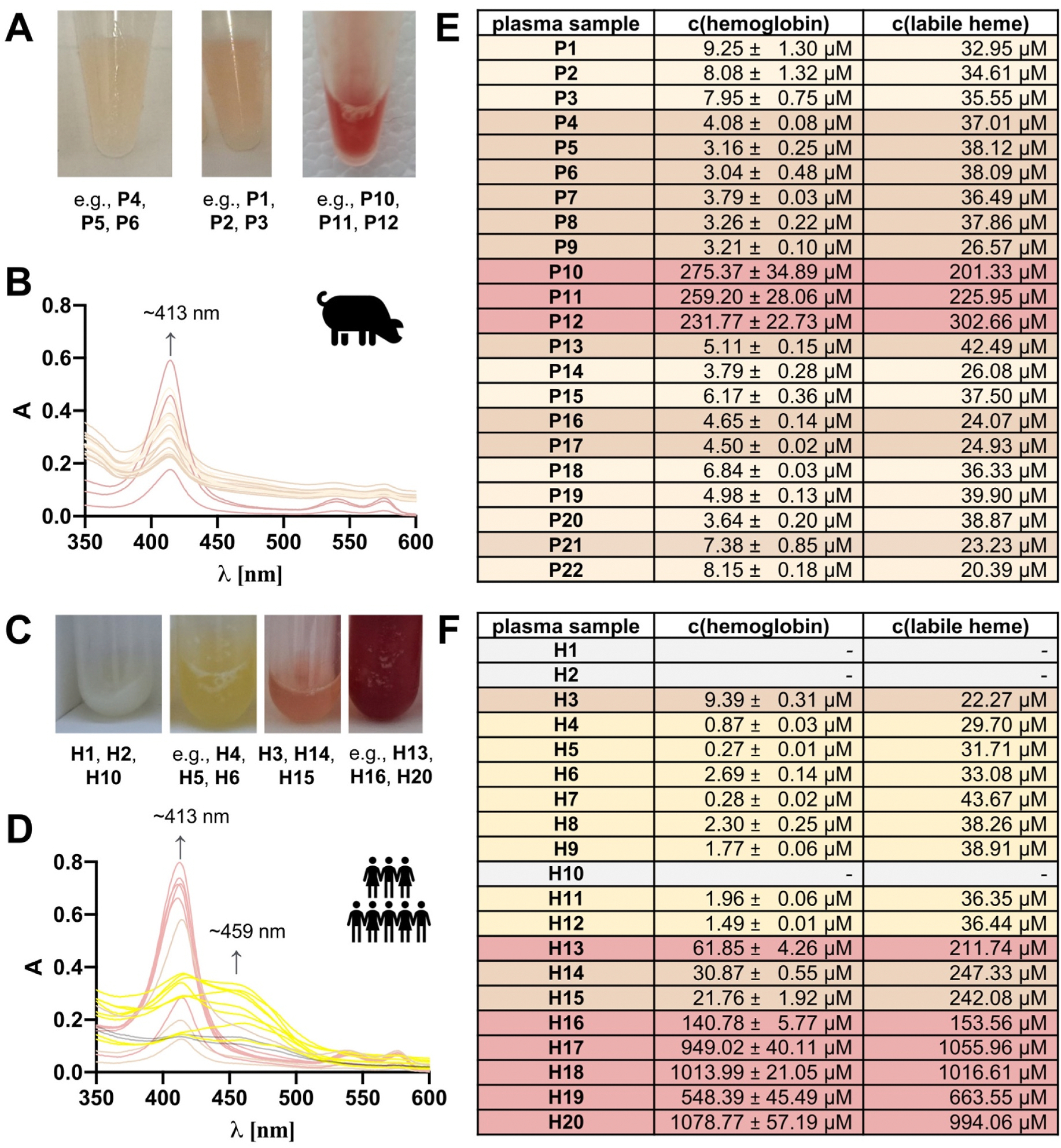
Heme and hemoglobin quantification from porcine and human plasma samples. (A) The porcine plasma samples **P1**–**P22** are characterized by a light brownish, light orange, or red color. Examples are given. (B) **P1**–**P22** show a clear absorbance maximum at ~413 nm to different extents. The spectra are colored analogous to the plasma sample. If required, the samples were diluted with PBS to obtain absorbance data <1.0. The dilution factors were in the range of 1–20. (C) The human plasma samples **H1**–**H20** had a pale, yellow, orange, or red color, as exemplified. (D) **H1**–**H20** show distinct absorbance spectra with maxima at ~413 nm and, in part, at ~459 nm. Spectra are colored analogous to the respective plasma sample. If required, the plasma samples were diluted with PBS to obtain absorbance data <1.0. The dilution factors were in the range of 1–72. (E) In the porcine plasma samples, hemoglobin was detected by the Harboe method in the range of ~3–280 μM, while the heme concentration was determined by Eq. 1 in the range of ~20–303 μM. (F) In the human plasma samples, hemoglobin was detected by the Harboe method in the range of ~0.3–1080 μM, while the heme concentration was determined in the range of ~22–1056 μM by applying Eq. 1.

A selection of 22 porcine and 20 human blood plasma samples was investigated for their hemoglobin and labile heme content by using the combination of the Harboe method and the Heme Assay Kit^®^ with Eq. 1 (Fig. 5). The plasma samples were of different hemolysis states as observed by distinct colors and absorbance spectra (Fig. 5A–D). Most of the samples were characterized by a spectrum with the prominent band at ~413 nm for hemoglobin (Fig. 5). In case of the yellow human plasma samples (**H4** – **H9**, **H11**, and **H12**) also a second maximum at ~450/460 nm was observed. The respective spectra and color of the samples were typical for the presence of bilirubin [31]. Furthermore, three plasma samples (**H1**, **H2**, and **H10**) were milky white and turbid (lipemic), which made hemoglobin and heme determination impossible. As expected, the color of the remaining samples correlated with the amount of hemoglobin detected with the Harboe method [16] (Fig. 5E and F). For the hemoglobin-rich (>100 μM), red-colored porcine and human plasma samples, also high levels of heme (~154–1056 μM) could be detected. The remaining porcine plasma samples contained ~3–10 μM hemoglobin, but also heme in the range of ~20–42 μM (Fig. 5E). Beyond these samples, the darker orange samples from patients showed similar results at higher level with ~9–31 μM hemoglobin and ~22–247 μM heme (Fig. 5F). Finally, the eight bilirubin-containing samples **H4** – **H9**, **H11**, and **H12** were all characterized by very low hemoglobin (~0.3–3 μM) and heme (~30–44 μM) levels.

## 4. Discussion

**Fig. 6.**
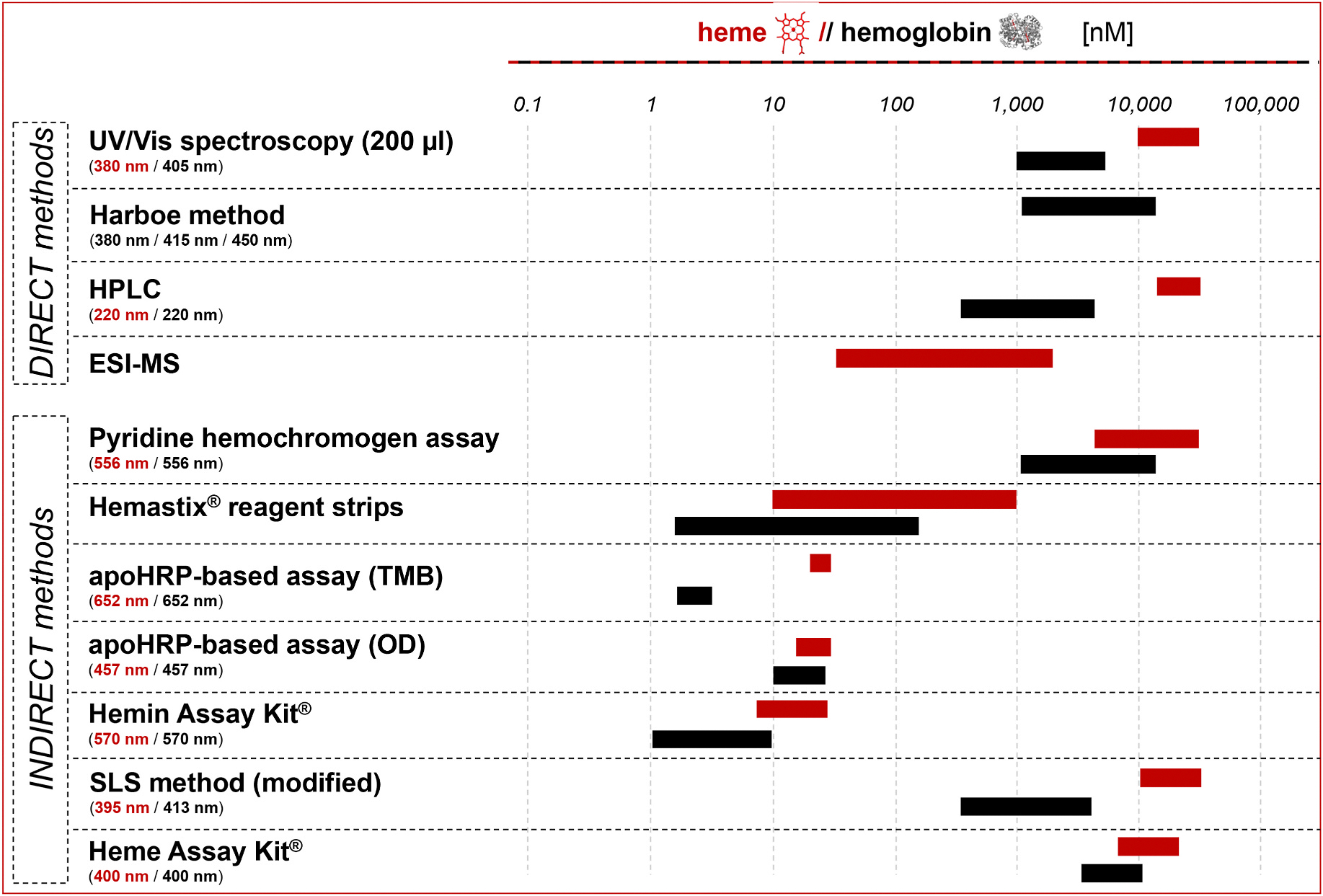
Overview of the heme and hemoglobin quantification ranges of direct and indirect methods.

In the past, enormous efforts were undertaken to establish heme quantification methods but failed to differentiate between hemoglobin-bound and labile heme [2,9,32]. In the present study, ten commonly used heme and hemoglobin quantification methods were analyzed with respect to their linearity, recovery, specificity/selectivity, and detection/quantitation limits. Noteworthy, these methods cover a quantification range of ~0.01–50 μM heme and ~0.001–10 μM hemoglobin (Fig. 6). Yet, while the Harboe method detected hemoglobin with acceptable recovery also in the presence of heme, none of the methods detected labile heme specifically. In contrast, the heme content of hemoglobin was underestimated, which even hampered total heme determination. This means, that e.g., the pyridine hemochromogen assay is not suitable for reliable total heme quantification, for which it has been applied [33]. HPLC analysis of hemoglobin revealed an average amount of six heme molecules per hemoglobin which exceeds the actual expected four heme molecules, which may be caused by additional heme in the hemoglobin samples due to transiently bound heme, as explored earlier [25]. The apoHRP-based assays showed the highest deviations and could not distinguish between hemoglobin-bound and labile heme, which is often not considered [34].

With respect to the required sample volume, the range of quantification, and the reliability, the Harboe method seems useful for hemoglobin quantification and the Heme Assay Kit^®^ for heme quantification. Due to the disturbing linear effect of hemoglobin concerning the Heme Assay Kit^®^, a combined approach with an adjusted equation was developed herein which allowed for the subtraction of the hemoglobin concentration determined by the Harboe method from the Heme Assay Kit^®^ result and showed the most reliable results for labile heme determination from hemoglobin-heme mixtures. This approach was thus applied to a series of 22 porcine and 20 human blood plasma samples. A variety of hemoglobin and heme levels and, thus, different hemolytic states could be observed that correlated with the sample color. In the hemoglobin-rich (>100 μM) samples, high labile heme levels up to ~1056 μM were detected. In some of the human plasma samples, hemoglobin was close to a concentration of 1 mM, which was a sign of massive intravascular hemolysis. In contrast, in samples with low hemoglobin (<10 μM), heme was determined in the range of ~20–44 μM. With respect to the hemoglobin concentration, some samples were non-hemolytic, since the hemoglobin concentration was <5 μM. In contrast, eight human plasma samples contained bilirubin, as observed by yellow color and the absorbance spectra. Thus, advanced intravascular hemolysis resulted in a massive degradation of hemoglobin to heme and the heme degradation product bilirubin. As such, hemoglobin and heme levels were very low in these samples.

The application of the combination of the Harboe method and the Heme Assay Kit^®^ with the presented equation is easy to use, requires low sample volume and enables fast performance. In the future, this technique may support estimation of labile heme levels and should be tested in large cohorts of patients suffering from hemolytic disorders. Additionally, the need for a novel technique for exact labile heme quantification is emphasized, which is required for clinical heme monitoring and understanding of hemolytic disorders on the molecular basis.

## Supplementary Material

Supp_mat

## Data Availability

Data will be made available on request.

## HIGHLIGHTS

- Heme initiates prothrombotic and proinflammatory conditions in hemolytic events.
- Heme and hemoglobin detection methods are evaluated by bioanalytical test criteria.
- Available methods cannot distinguish between hemoglobin-bound heme and labile heme.
- A strategy for heme detection in blood plasma samples is provided.
