# Supplementary material for: Quantitative analysis of heme and hemoglobin for the detection of intravascular hemolysis
Source: Anal Chim Acta. Author manuscript; Available in PMC 2024 Dec 8. (PMC11625178; doi:10.1016/j.aca.2024.342766)
Supplement: Supp_mat [file NIHMS2037623-supplement-Supp_mat.pdf]

Quantitative Analysis of Heme and Hemoglobin for the Detection of Intravascular Hemolysis

Marie-T. Hopp[a,b], Sonali M. Vaidya[a], Karina M. Grimmig[a], Lasse J. Strudthoff[c], Johanna C. Clauser[c], Xiaojing Yuan[d], Sneha Singh[e], Jens Müller[e], Johannes Oldenburg[e], Iqbal Hamza[d], and Diana Imhof[a,*]

[a] Pharmaceutical Biochemistry and Bioanalytics, Pharmaceutical Institute, University of Bonn, D-53121 Bonn, Germany

[b] Department of Chemistry, Institute for Integrated Natural Sciences, University of Koblenz, D-56070 Koblenz, Germany

[c] Department of Cardiovascular Engineering, Institute of Applied Medical Engineering, Medical Faculty RWTH Aachen University, D-52074 Aachen, Germany

[d] Center for Blood Oxygen Transport and Hemostasis, Department of Pediatrics, University of Maryland School of Medicine, US-21201 Baltimore, MD, USA

[e] Institute of Experimental Hematology and Transfusion Medicine, University Hospital Bonn, D-53127 Bonn, Germany

---

Supplementary material

1. Supplementary methods

1.1. Reagents and general procedures. Hemin, herein termed "heme", was obtained from Frontier Scientific (Logan, UT, USA). Heme (1 mM) was prepared freshly in 30 mM sodium hydroxide solution. The exact concentration was determined by applying the molar extinction coefficient ε_{398} = 32.6 mM^{-1} cm^{-1} (100 mM HEPES, pH 7.0) [1]. Accordingly, the alkaline stock solution was diluted with phosphate buffer saline (1x PBS, pH 7.4) for further use. As analyzed before [2], the lyophilized human hemoglobin (Sigma-Aldrich, St. Louis, MO, USA) consists of methemoglobin and was characterized by native and SDS-PAGE. Following general practice, the term "hemoglobin" is used herein for "methemoglobin". Hemoglobin (1 mM) was dissolved in 1x PBS (pH 7.4) and further diluted with PBS to the desired concentrations. Heme-hemoglobin mixtures were incubated for 30 min at room temperature prior to use. The Heme Assay Kit® (MAK316) and the Hemin Assay Kit® (MAK036) were purchased from Sigma-Aldrich (St. Louis, MO, USA). Hemastix® reagent test strips were obtained from Siemens Healthineers (Erlangen, Germany). According to Lambert-Beer's law [3], data were acquired within the absorbance range of 0.1 to 0.8. In exceptional cases values up to 1.0 were considered if linearity was given. For absorbance measurements of plasma samples, the ideal range of 0.2-0.5 was kept, if possible. All UV/Vis measurements were performed on a Multiskan GO spectrophotometer (Thermo Fisher Scientific, Waltham, MA, USA).

1.2. Characterization of hemoglobin samples. Hemoglobin was analyzed by MALDI-TOF-MS and native PAGE. MALDI-TOF-MS analysis of hemoglobin was performed as described before [2, 4], using 2,5-dihydroxyacetophenone as matrix. Further, 15 µl hemoglobin (1 mg/ml and/or 10 mg/ml in 1x PBS, pH 7.4) was applied and separated on a precast 4-16 % Bis-Tris gel (Invitrogen NativePAGETM, ThermoFisher Scientific, Waltham, MA, USA) in a XCell SureLock Mini-Cell electrophoresis system. The gel was resolved at 180 V under continuous cooling on ice. In addition, hemoglobin and its separated peaks from HPLC analysis were examined by SDS-PAGE using a 10 % separation gel. With both, native and SDS-PAGE, staining was performed overnight with colloidal Coomassie blue (Figure 3B, Figure S9).

1.3. Plasma sample preparation. Human citrated plasma samples were available from leftover specimens obtained for routine coagulation analysis. Samples were selected according to obvious signs of hemolysis (none too pronounced) and pipetted into numbered tubes without traceability to patients. The material was stored at ≤ -70°C until analysis. For porcine plasma preparation, 500 ml of whole blood was recirculated at controlled 4.5 lpm for up to 300 min in a miniaturized circuit comprising an HLS 7.0 oxygenator (Maquet Cardiopulmonary GmbH, Getinge SE, Rastatt, Germany), an elastic silicone reservoir (self-made design, Elastosil 620 Silicone, Wacker Chemie AG, Munich, Germany) and 12 cm of 3/8" ECC tubing (Raumedic AG, Helmbrechts, Germany). Different to commercially available HLS devices, the blood leading components of the tested devices were uncoated. The polymethylpentene fiber membranes within the oxygenator have a blood contacting surface of 1.8 m^2. Immediately after the collection of the blood, 100 ml of 0.9 % NaCl (B. Braun, Melsungen, Germany), 1.8 ml/l Gentamicin (Ratiopharm, Ulm, Germany), 1.2 ml/l Glucose and 2.000 IE/l Clexane (Sanofi SA, Paris, France) were added. During degressively distributed time points, blood samples were drawn via a three-valve-cock (Discofix C 3SC, B. Braun, Melsungen, Germany), citrated 10:1 using 3.131 % sodium citrate (Eifelfango GmbH & Co. KG, Bad Neuenahr-Ahrweiler, Germany), rested in a dark chamber on a vacillating rocking shaker for 15 min and centrifuged at 4.000 g for 15 min. The resulting platelet-poor plasma was frozen in aliquots at -80°C. The low anticoagulation dosage allowed spontaneous thrombus or pseudomembrane formation on the oxygenator fibers. To keep the flow constant, the integrated pump increases its rotational speed with increasing pressure drop over the oxygenator. With increasing rotational speed, commonly hemolysis increases as well. Upon reaching the functional limit of the pump drive at 5,000 rpm (Maquet Cardiopulmonary GmbH, Getinge SE, Rastatt, Germany), the experiments were terminated before 300 min. For every experiment day, two identical setups with blood from the same donor pig were run synchronously.
The plasma samples were frozen at -80 °C and thawed at room temperature before use. If required, the samples were diluted using 1x PBS, pH 7.4.

1.4. ESI-MS. Heme (39-1250 nM) was prepared in 50 % acetonitrile/water with 0.1 % TFA. The analyte solutions (20 µl) were injected into a LC (UltiMate 3000, Thermo Fisher Scientific) coupled to an ESI-MS system (micrOTOF Q-III, Bruker Daltonics

GmbH, Bremen, Germany). An isocratic solvent system was applied for 90 seconds, using 0.1 % acetic acid in acetonitrile as the eluent and a flow rate of 0.3 ml/min. The analyte was directly transferred to the ESI source. MS signals were acquired in the positive mode with a capillary voltage of 4.5 kV and an end plate offset of 500 V (Supplemental Table 1). Spectra were inspected with Data Analysis 2.1 (Bruker Daltonics GmbH). To generate the heme standard curve, the AUC of the mass peaks at m/z 616.177, derived from the accurate quantifier ion mass, was integrated from the extracted ion chromatogram (EIC) and plotted by using QuantAnalysis 2.1 (Bruker Daltonics GmbH).

1.5. ApoHRP-based assay with TMB. Heme (33.3-42.9 nM) and hemoglobin (2.9-5.2 nM) solutions were used, following a modified protocol derived from Atamna et al. (2015) [4, 5]. In brief, 40 µM apoHRP was mixed 1:1 with the analyte, diluted 1:5 in PBS and incubated for 10 min on ice. Subsequently, 10 µl of this mixture was combined with substrate solution (200 µl), containing a 1:1 mixture of 83 µM 3,3',5,5'-tetramethylbenzidine (TMB)/0.2 % 6 N HCl in water and 107 µM H2O2 in 0.1 M citric buffer (pH 5.0). The absorbance at 652 nm was recorded every 5 seconds for 40 min. As controls, the analyte and apoHRP were substituted by PBS. For the evaluation, the absorbance of the controls was subtracted. The activity of the reconstituted HRP was evaluated as the absorbance change per minute. Mixtures of heme (33 nM and 38 nM) and hemoglobin (3.3 and 3.8 nM) were analyzed as well.

1.6. ApoHRP-based assay with o-dianisidine. For this approach, solutions of heme (final: 23.8-41.7 nM) and hemoglobin (final: 9.5-35.7 nM) were used to generate the respective standard curves. The substrate solution was prepared using a mixture of 0.83 mM o-dianisidine dihydrochloride and 1.07 mM hydrogen peroxide in 0.1 M phosphate buffer (pH 6.0) and the absorbance was recorded at 457 nm.

1.7. Hemin Assay Kit®. According to the manufacturer's instructions (Sigma-Aldrich, St. Louis, MO, USA), 43 µl heme solution (final concentration: 2 – 32 nM) was mixed with 2 µl "enzyme mix" *, 3 µl "hemin substrate" *, and 2 µl "hemin probe" * (*reagents from the kit), yielding a total volume of 50 µl on a 96-well microtiter plate. The same was applied for hemoglobin standard solutions (final concentration: 0.5 – 12 nM). Immediately after the addition of the hemin probe, the absorbance at 570 nm was

monitored every 5 s over 40 min. The results were evaluated similarly to the apoHRP-based assay (see section 2.4.5). The enzyme mix was analyzed by means of SDS-PAGE and MALDI-TOF-MS (Figure S4), the assay buffer was measured by ESI-MS (Figure S5). Due to the large, overlapping error bars within the standard heme and hemoglobin curves, only one addition experiment of heme (20 nM) and hemoglobin (5 nM) together was performed.

1.8. Heme Assay Kit®. Following the manufacturer's instructions, 50 µl of heme (8-32 µM) were mixed with 200 µl kit reagent on a microtiter plate. After 5 min incubation, the absorbance was measured at 400 nm. The same procedure was carried out with hemoglobin (0.5-10 µM) and mixtures of heme (final: 5 µM, 10 µM, 15 µM) and hemoglobin (final: 2 µM, 5 µM).

2. Supplementary figures

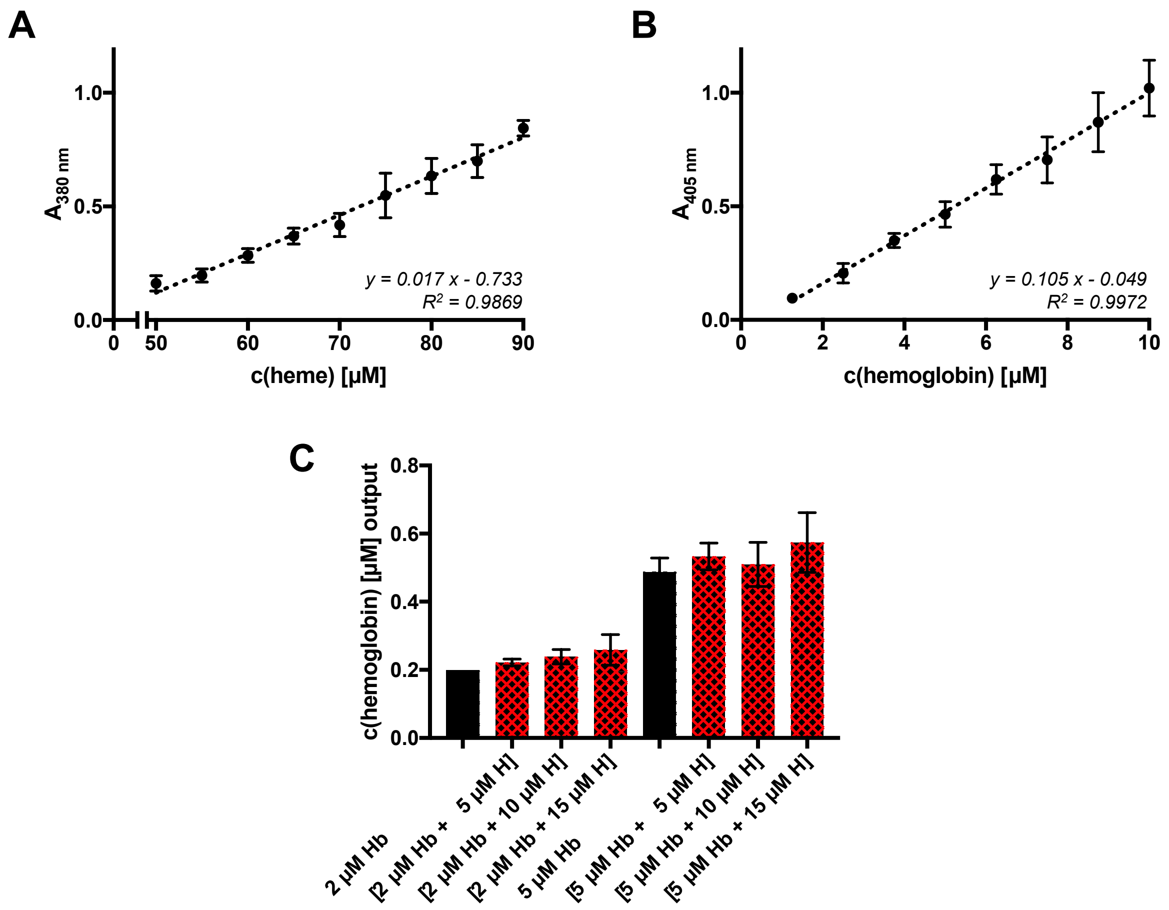

Fig. S1. (A) Heme and (B) hemoglobin calibration curves for the in-house UV/Vis spectroscopic method at 380 nm and 405 nm, respectively, in 1x PBS (pH 7.4) with a volume of 100 µl in a 96-well microtiter plate. (C) Addition of heme (5 µM, 10 µM, and 15 µM) to hemoglobin (2 µM and 5 µM) has no significant effect on the absorbance at 405 nm and, thus, hemoglobin determination.

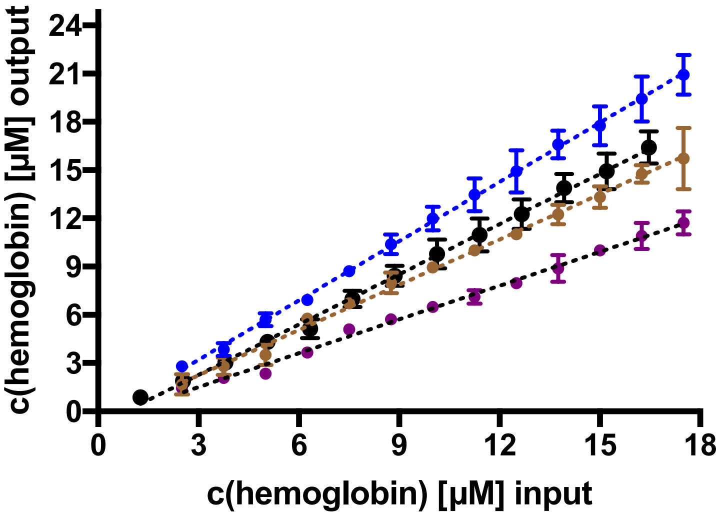

Fig. S2. Comparison of hemoglobin recovery in PBS (black), in water with 0.1 % Na_2CO_3 (purple), in PBS with 0.1 % Na_2CO_3 (brown), and in 288 µM Tris-HCl buffer (blue). While the usage of Tris-HCl buffer leads to the overestimation of the hemoglobin concentration, the addition of 0.1 % Na_2CO_3 to PBS results in a slight underestimation of hemoglobin and the addition of 0.1 % Na_2CO_3 to water yields a massive underestimation of the hemoglobin concentration.

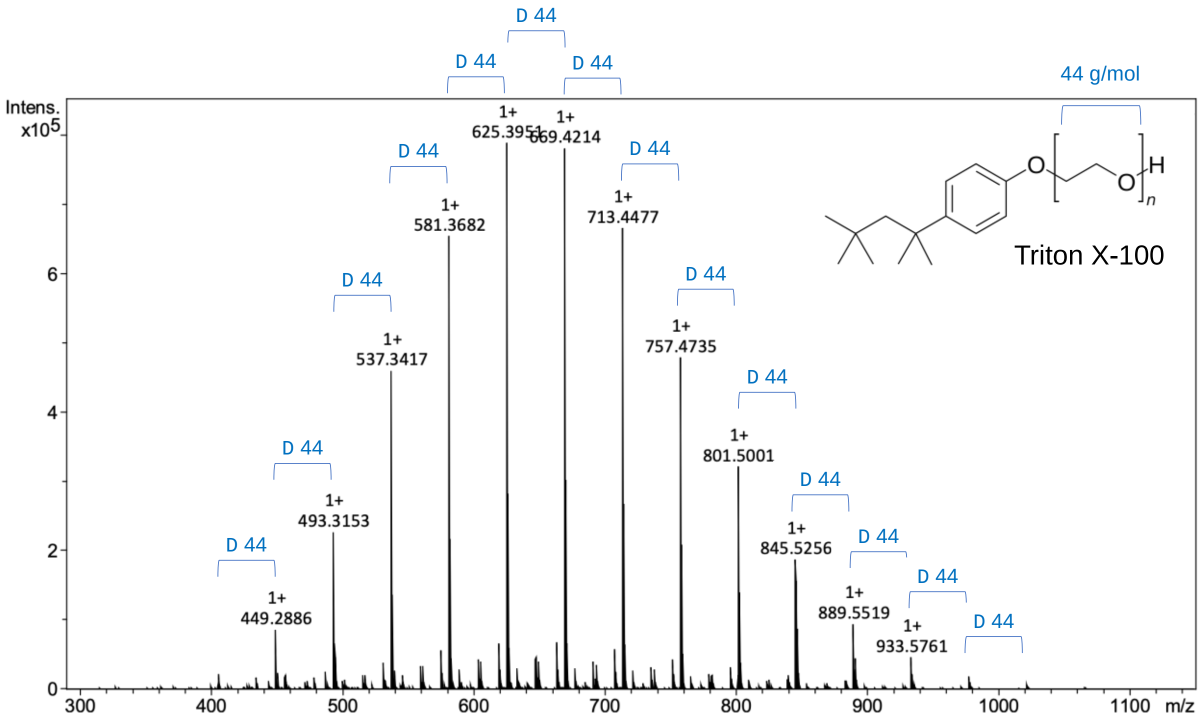

Fig. S3. Analytical characterization of the Heme Assay Kit® reagent. ESI-MS analysis of the reagent revealed the typical MS spectrum pattern of the non-ionic surfactant Triton X-100 [6].

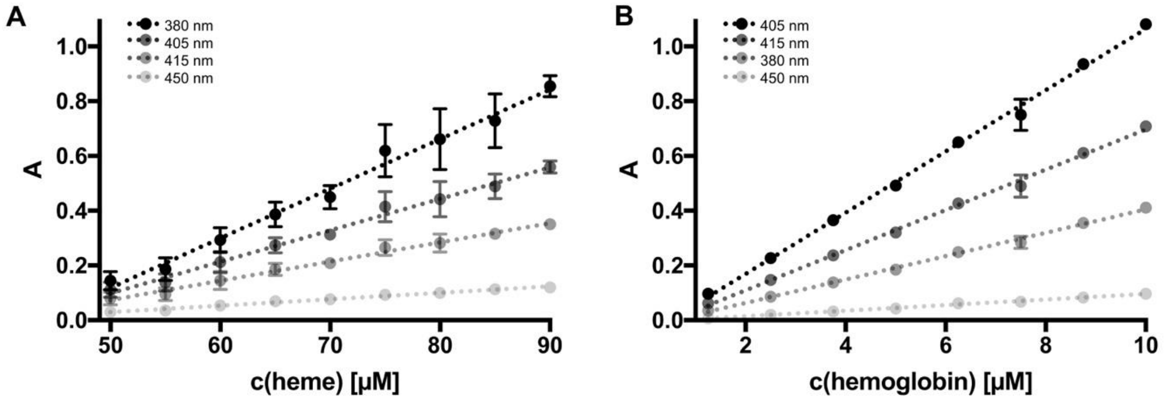

Fig. S4. Comparison of the absorbance level of (A) heme and (B) hemoglobin in 1x PBS (pH 7.4) at different wavelengths and a volume of 100 µl. The wavelengths include the actual absorbance maxima, i.e., 380 nm for heme and 405 nm for hemoglobin, as well as the other wavelengths used for the Harboe method (415 nm and 450 nm).

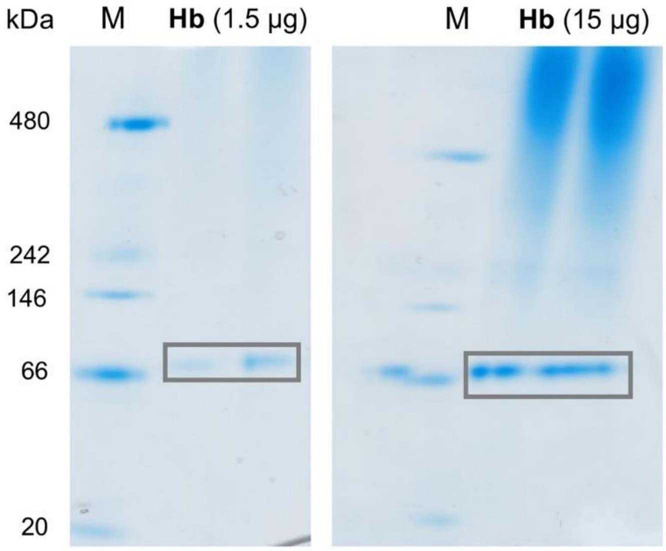

Fig. S5. Native PAGE of hemoglobin in 1x PBS, pH 7.4, reconstituted commercially purchased hemoglobin (Sigma-Aldrich, St. Louis, MO, USA).

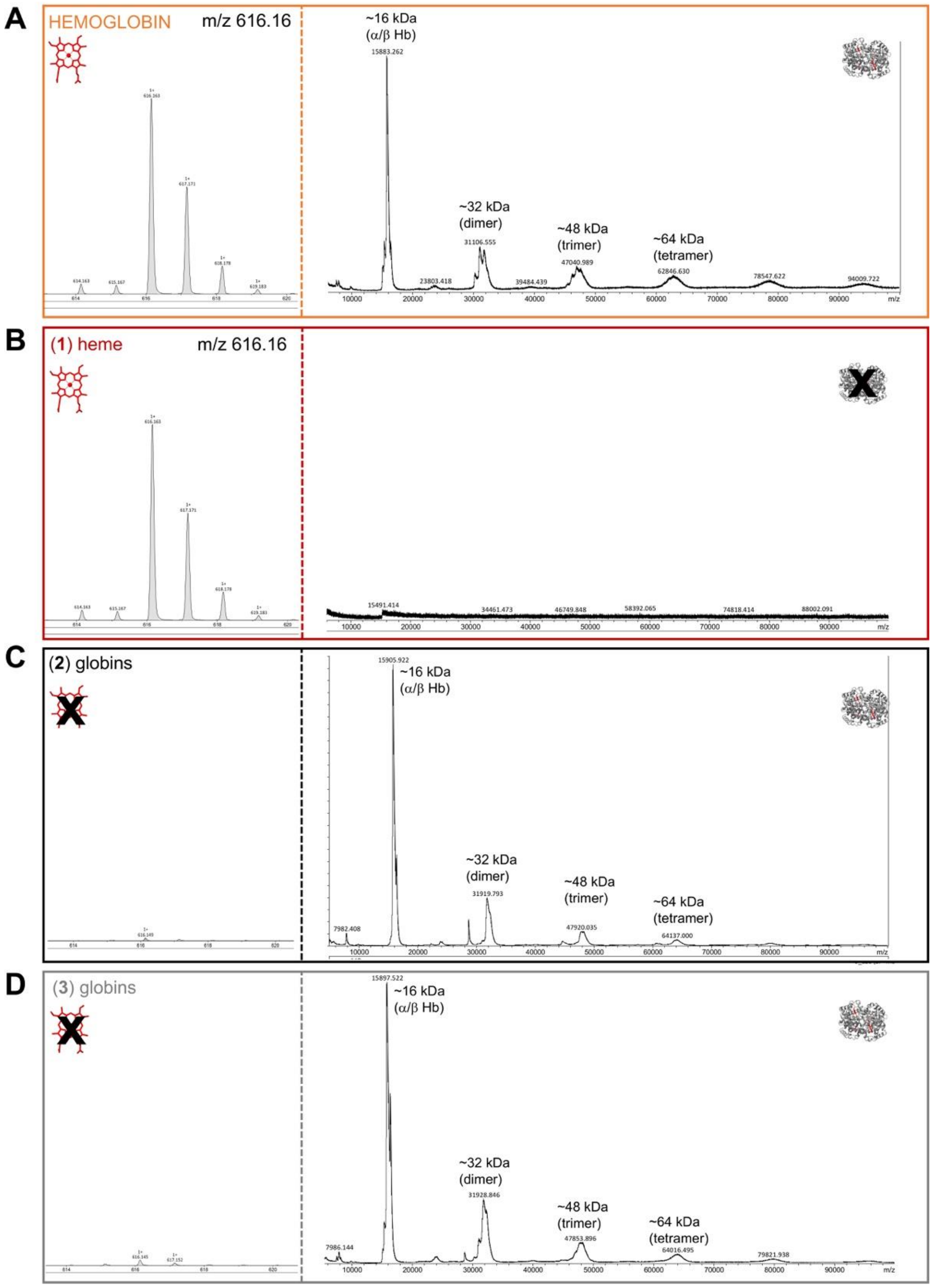

Fig. S6. MALDI-MS spectra of hemoglobin (M_W theor.: 64.5 kDa) and HPLC-separated fractions of hemoglobin. (A) Commercially purchased hemoglobin was spotted once with HCCA (for heme analysis) and once with DHAP (for protein analysis) as a matrix and analyzed in the lower and higher m/z range, respectively, revealing an intense signal for heme (M_W theor.: 616.18 g/mol) (left) and the globin chains (right). (B-D) Hemoglobin was analyzed after separation by HPLC: (B) In the first fraction, an intense peak for heme but no protein was found. (C) In the second fraction, no peak for heme (left) but a high-intensity peak for the globin chains (M_W ~16 kDa, ~32 kDa, ~48 kDa, ~64 kDa) (right) was detected. (D) The third fraction revealed a similar pattern as observed for the second fraction.

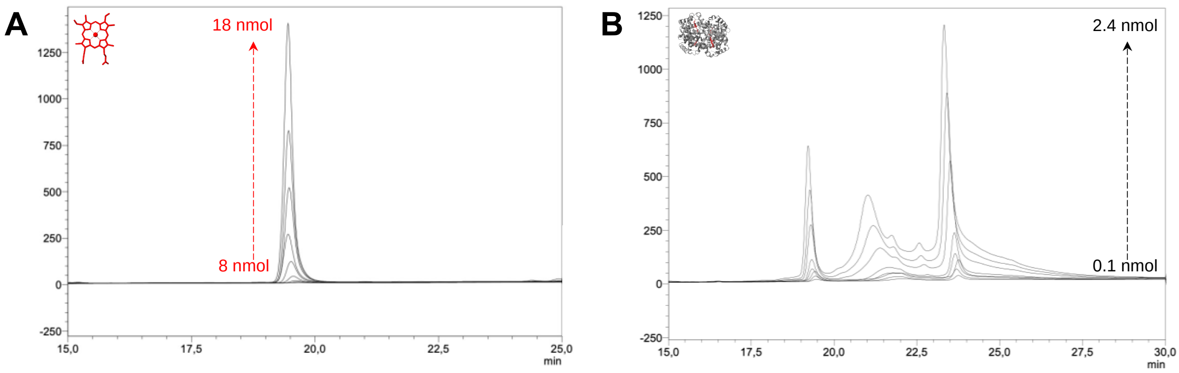

Fig. S7. Overlay of chromatograms of heme (8 - 18 nmol) and hemoglobin (0.1 - 2.4 nmol) as recorded at 220 nm.

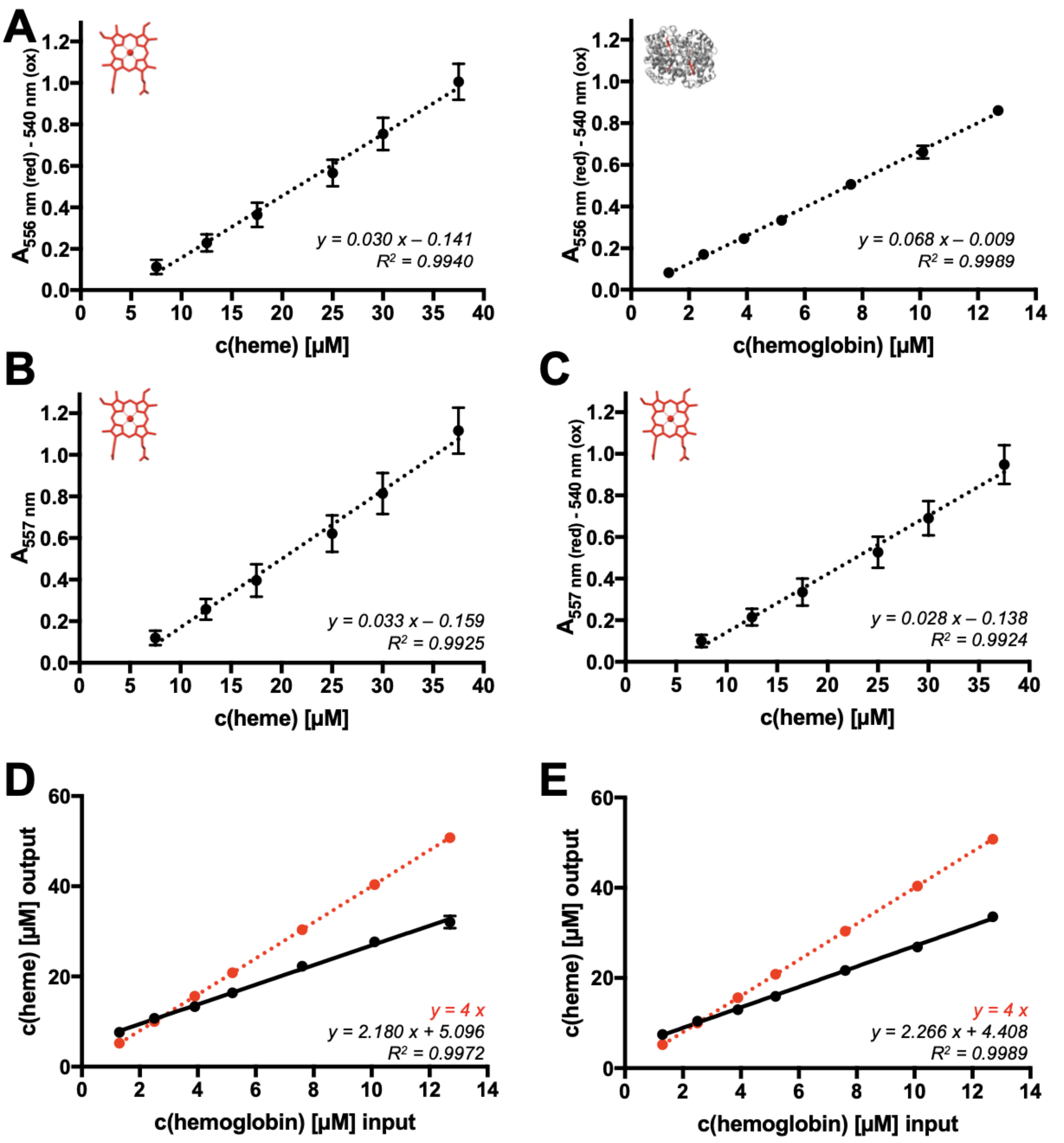

Fig. S8. Alternative evaluation of the pyridine hemochromogen assay. (A) Standard heme (left) and hemoglobin (right) curves were generated by plotting the absorbance difference of the reduced (556 nm) and the oxidized (540 nm) sample. For comparison to earlier reported extinction coefficients, the heme standard curve for (B) 557 nm (reduced sample) as well as (C) the respective absorbance difference of the reduced (557 nm) and oxidized (540 nm) sample was analyzed. Slightly different extinction coefficients (e_{557} = 32.91 mM^{-1} cm^{-1} and $e_{557\ (red)\ -\ 540\ (ox)}$ = 28.03 mM^{-1} cm^{-1}) were obtained in comparison to the earlier reported ones (e_{557} = 34.70 mM^{-1} cm^{-1} and $e_{557\ (red)\ -\ 540\ (ox)}$ = 23.98 mM^{-1} cm^{-1}) [7,8]. (D) When evaluating the standard hemoglobin solutions with the heme equation (at 556 nm), for each hemoglobin molecule ~2.18 heme molecules (black) instead of four (red) are detected. (E) When evaluating the standard hemoglobin solutions with the heme equation (difference at 556 nm (reduced sample) and 540 nm (oxidized sample)), for each hemoglobin molecule ~2.27 heme molecules (black) instead of four (red) are detected.

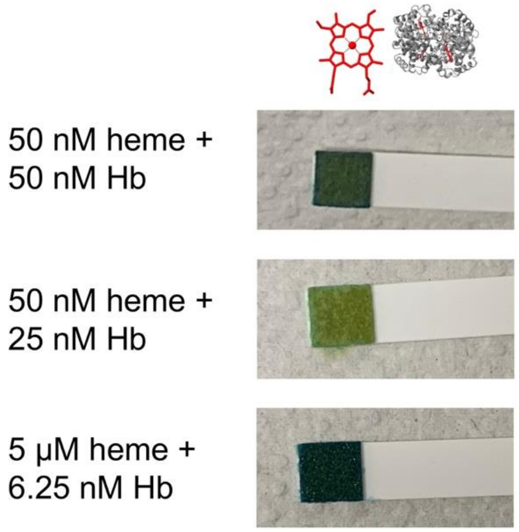

Fig. S9. Application of Hemastix® test strips for mixtures of heme and hemoglobin.

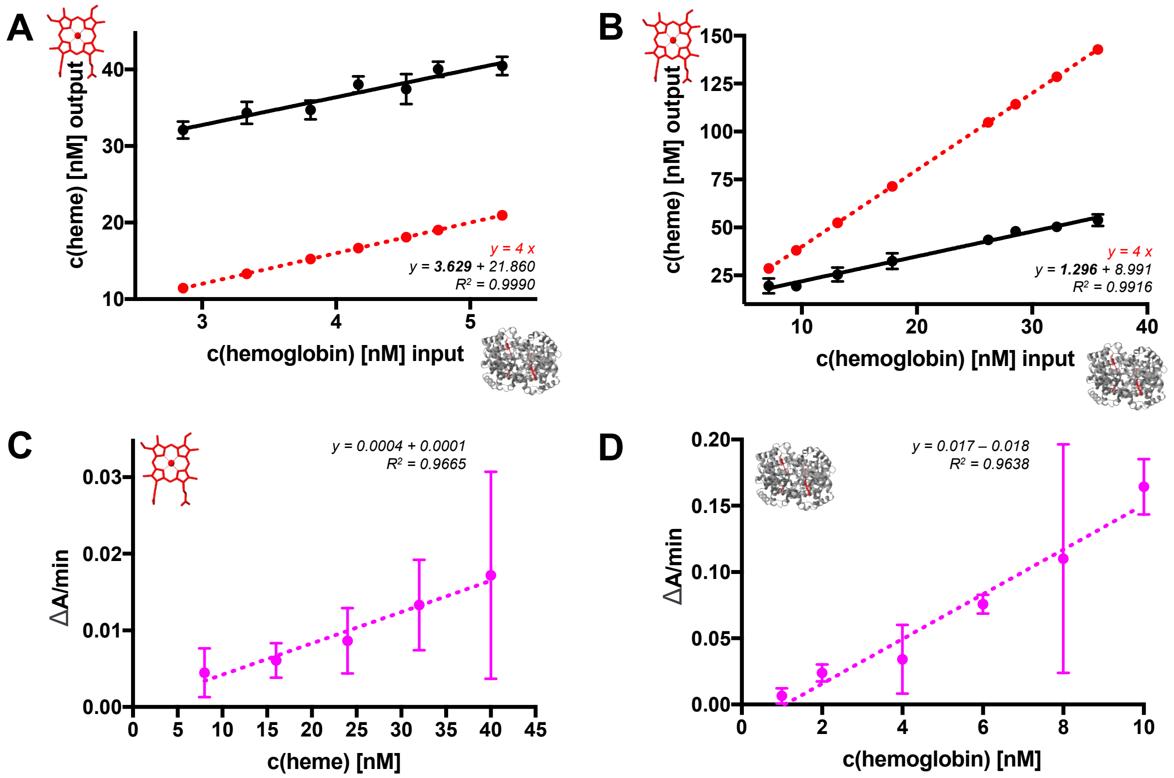

Fig. S10. Additional information about the apoHRP-based assay systems. (A) Recalculation of the heme concentration from hemoglobin solutions via the apoHRP-based assay with TMB as the substrate reveals a massive overestimation. (B) With *o*-dianisidine, approximately 1.3 heme molecules are detected per hemoglobin molecule. (C) With the Hemin Assay Kit®, the concentrations (8 – 40 nM), as suggested by the manufacturer, were tested, showing large standard deviations. (D) Concentration determination in the range of 1 – 10 nM of hemoglobin detected with the same assay.

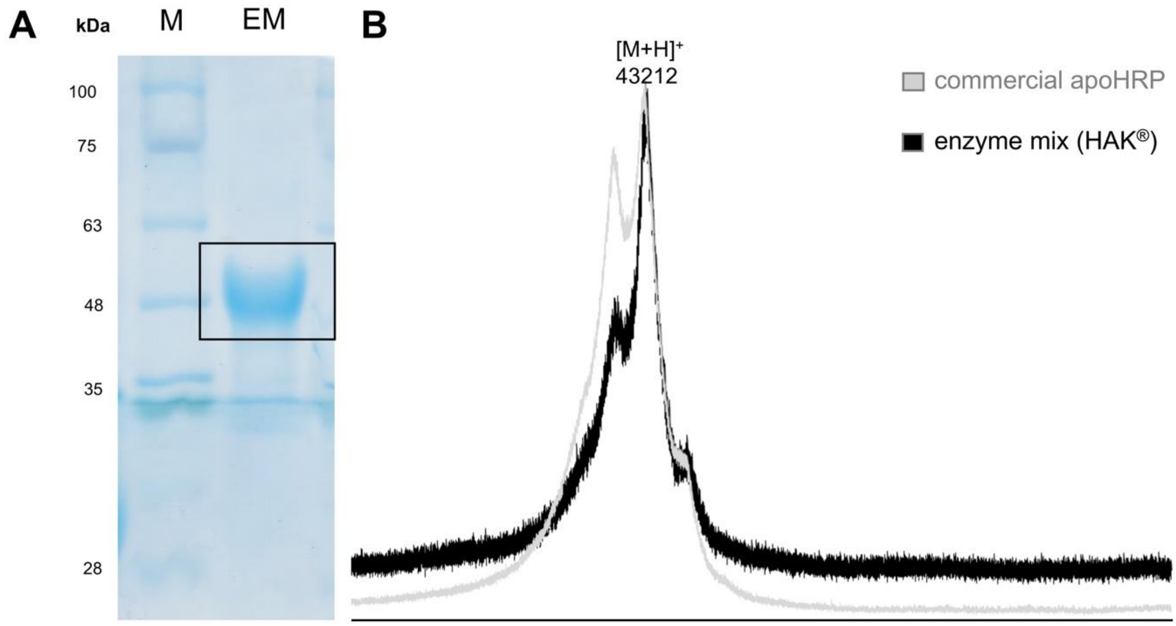

Fig. S11. Analytical characterization of the Hemin Assay Kit® enzyme mix. (A) SDS-PAGE analysis under non-reducing conditions shows that the enzyme mix contains a major component with a molecular weight of ~45 kDa. (B) MALDI-TOF-MS analysis of the enzyme mix in the linear mode supports the presence of only one component with m/z 43212 (black). The obtained pattern is equal to the spectrum obtained with commercial apoHRP (grey). M, marker; EM, enzyme mix.

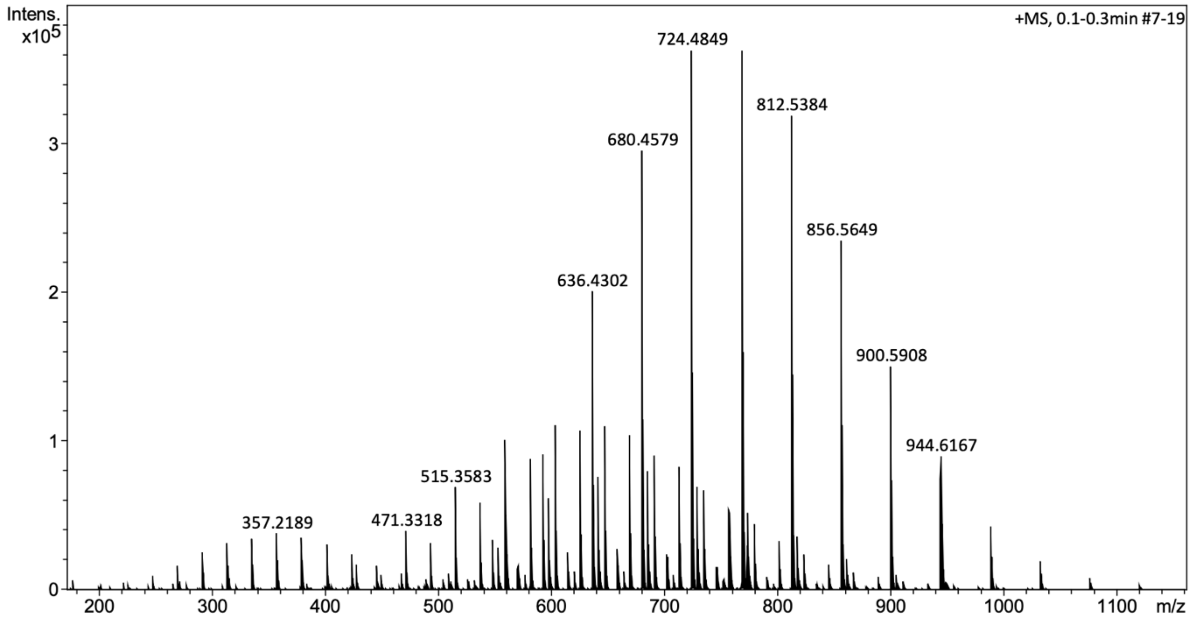

Fig. S12. Analytical characterization of the Hemin Assay Kit® buffer. ESI-MS analysis of the buffer reveals three ingredients characterized by patterns with mass differences (Δ) of 44 g/mol, accounting for the presence of PEG and/or related compounds such as Tritons and Tween.

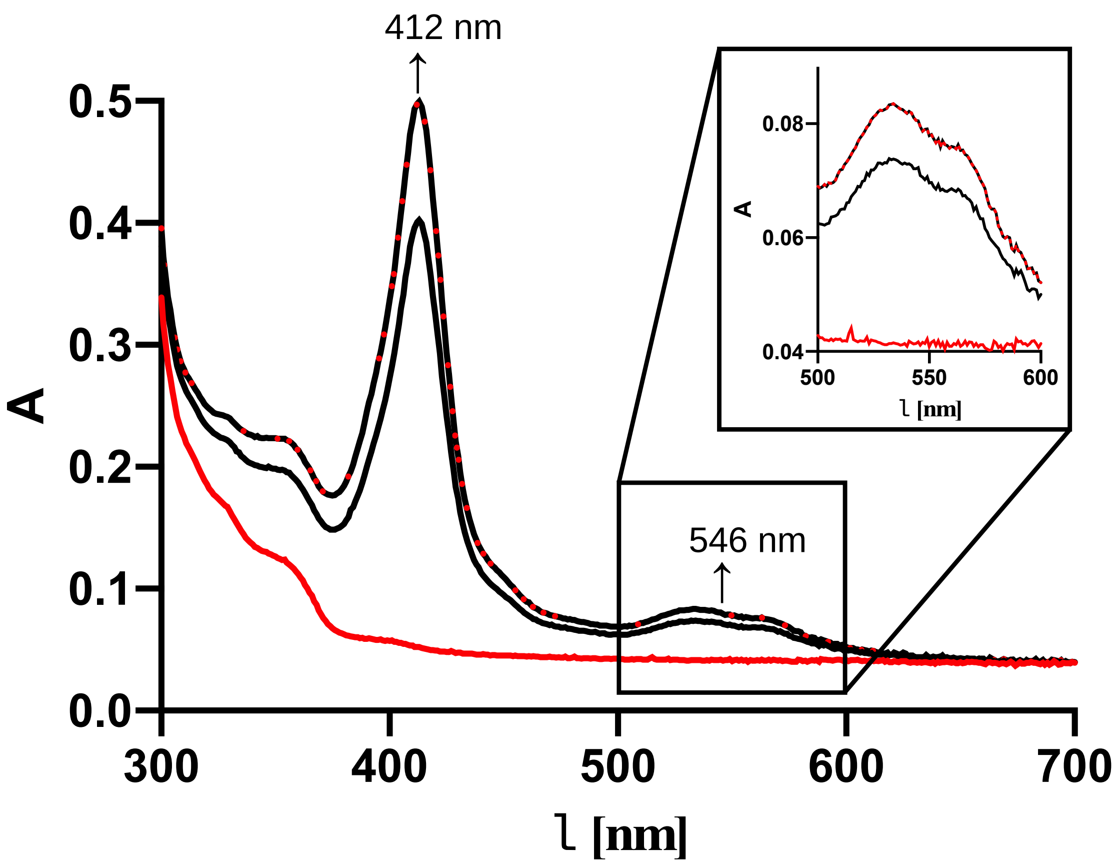

Fig. S13. Absorbance spectra (300 – 700 nm) of heme (10 µM; red) and hemoglobin (10 µM; black) and the heme (10 µM) - hemoglobin (10 µM) mixture (dashed line) upon addition of the SLS assay reagent. The method quantifies hemoglobin by using its absorbance at 546 nm in the presence of SLS.

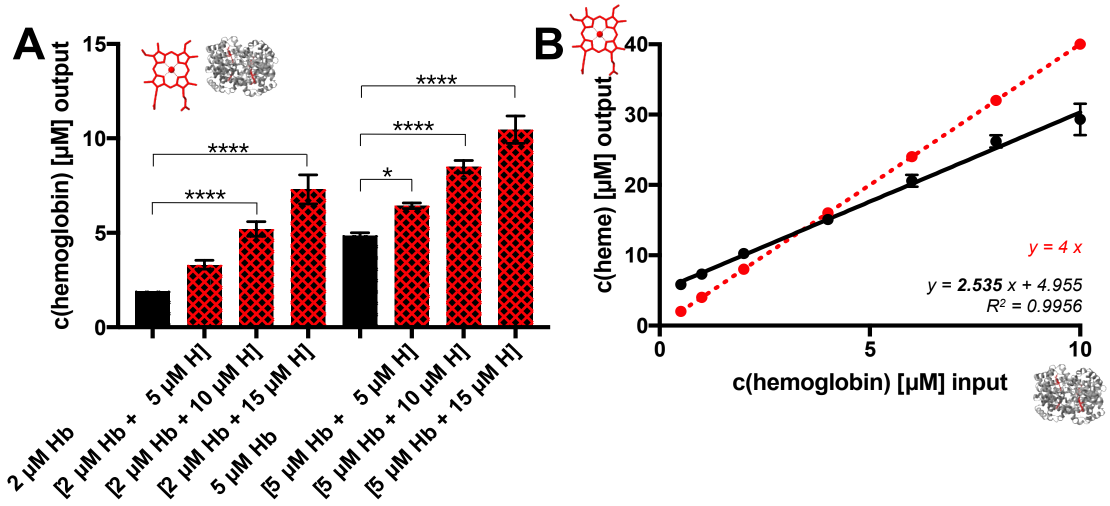

Fig. S14. (A) Addition of heme (5 µM, 10 µM, and 15 µM) to hemoglobin (2 µM and 5 µM) and its impact on the hemoglobin recovery using the Heme Assay Kit®. (B) Evaluation of the heme concentration in a hemoglobin solution via the heme calibration curve suggests a ratio of ~2.54 heme molecules per hemoglobin.

3. Supplementary Tables

Tab. S1. Conditions for the ESI-MS-based heme quantification.

Parameter	Value
**ESI interface**	
scan range	m/z 50 – 2,000
capillary voltage	4.5 kV
nebulizer gas	2.2 bar
dry gas	10 l/min; 200°C
end plate offset	500 V
**Transfer voltage**	
funnel 1 and 2	200 Vpp
hexapole radio frequency voltage	100 Vpp
**Quadrupole**	
ion energy	5.0 eV
**Collision cell**	
collision energy	8.0 eV
collision RF	200 Vpp
transfer time	60 µs

Tab. S2. Overview of the calculated estimated limit of detection (LOD) and LOQ (limit of quantitation) according to the ICH guidelines [8]. Hb, hemoglobin; Sy, standard deviation of the response; S, slope of the calibration curve.

	**LOD** (calculated as 3.3 Sy/S according to ICH [8])	**LOQ** (calculated as 10 Sy/S according to ICH [8])
**UV/Vis** (in-house; 200 µl)	3.30 µM (heme)<br>0.43 µM (hemoglobin)	10.01 µM (heme)<br>1.31 µM (hemoglobin)
**Harboe method** (PBS)	0.46 µM (hemoglobin)	1.38 µM (hemoglobin)
**HPLC**	2.65 nmol (heme)<br>0.11 nmol (hemoglobin)	8.04 nmol (heme)<br>0.32 nmol (hemoglobin)
**ESI-MS**	0.96 pmol (heme)	2.91 pmol (heme)
**Pyridine hemochromogen assay** (556 nm)	3.51 µM (heme)<br>0.34 µM (hemoglobin)	10.64 µM (heme)<br>1.02 µM (hemoglobin)
**apoHRP-based assay** (TMB)	12.54 nM (heme)<br>1.65 nM (hemoglobin)	38.00 nM (heme)<br>-
**apoHRP-based assay** (*o*-dianisidine)	13.94 nM (heme)<br>3.45 nM (hemoglobin)	-<br>10.45 nM (hemoglobin)
**Hemin Assay Kit®**	9.41 nM (heme)<br>1.94 nM (hemoglobin)	28.51 nM (heme)<br>5.88 nM (hemoglobin)
**SLS method** (modified)	3.74 µM (heme)<br>0.23 µM (hemoglobin)	11.33 µM (heme)<br>0.71 µM (hemoglobin)
**Heme Assay Kit®**	1.93 µM (heme)<br>0.40 µM (hemoglobin)	-<br>1.22 µM (hemoglobin)

Tab. S3. Heme and hemoglobin recovery with the UV/Vis spectroscopic method (200 µl sample volume) applying the equation of the calibration curve for heme ($y = 0.029\ x - 0.170$) and hemoglobin ($y = 0.158\ x - 0.007$) to three dilution series. Recoveries with deviations of <15 % from 100 % recovery are highlighted in green. Recoveries with deviations of >15 % from 100 % recovery are marked in red.

Input concentration	Mean output concentration	Mean recovery rate
**Heme determination with *y* = 0.029 *x* – 0.170**		
10 µM	10.72 ± 1.34 µM	107.22 ± 13.41 %
15 µM	14.41 ± 2.28 µM	96.05 ± 15.17 %
20 µM	20.63 ± 2.88 µM	103.16 ± 14.38 %
25 µM	23.34 ± 4.59 µM	93.38 ± 18.37 %
30 µM	30.33 ± 5.16 µM	101.11 ± 17.20 %
35 µM	35.84 ± 5.99 µM	102.40 ± 17.11 %
40 µM	39.87 ± 4.72 µM	99.68 ± 11.79 %
		⌀ 100.43 ± 15.35%
**Hemoglobin determination with *y* = 0.158 *x* – 0.007**		
1 µM	1.01 ± 0.09 µM	101.27 ± 8.51 %
2 µM	1.98 ± 0.20 µM	99.05 ± 10.15 %
3 µM	2.99 ± 0.33 µM	99.51 ± 11.01 %
4 µM	4.32 ± 1.14 µM	108.02 ± 28.56 %
5 µM	5.02 ± 0.86 µM	100.42 ± 17.22 %
6 µM	6.15 ± 0.39 µM	102.53 ± 6.45 %
7 µM	6.89 ± 0.54 µM	98.40 ± 7.76 %
		⌀ 101.31 ± 12.81%

Tab. S4. Heme and hemoglobin recovery with the UV/Vis spectroscopic method (100 µl sample volume) applying the respective equations derived from the calibration curves of heme (y = 0.0171 x – 0.733) and hemoglobin (y = 0.1049 x – 0.049) to three dilution series. A path length of 3 mm was considered. Recoveries with deviations of <15 % from 100 % recovery are highlighted in green. Recoveries with deviations of >15 % from 100 % recovery are marked in red.

Input concentration	Mean output concentration	Mean recovery rate
**Heme determination with *y* = 0.0171 *x* – 0.733**		
50 µM	51.33 ± 1.94 µM	102.66 ± 3.89 %
55 µM	53.80 ± 2.46 µM	97.82 ± 4.47 %
60 µM	59.98 ± 2.65 µM	99.97 ± 4.42 %
65 µM	65.48 ± 2.62 µM	100.73 ± 4.04 %
70 µM	69.18 ± 2.47 µM	98.83 ± 3.54 %
75 µM	79.07 ± 5.57 µM	105.43 ± 7.42 %
80 µM	81.55 ± 6.50 µM	101.93 ± 8.12 %
85 µM	85.45 ± 5.74 µM	100.53 ± 6.76 %
90 µM	92.84 ± 2.29 µM	103.15 ± 2.55 %
		⌀ 101.23 ± 5.02%
**Hemoglobin determination with *y* = 0.1049 *x* – 0.049**		
1.25 µM	1.38 ± 0.05 µM	110.78 ± 3.78 %
2.5 µM	2.63 ± 0.07 µM	105.12 ± 2.89 %
3.75 µM	3.95 ± 0.06 µM	105.24 ± 1.56 %
5 µM	5.15 ± 0.15 µM	103.07 ± 2.91 %
6.25 µM	6.67 ± 0.12 µM	106.69 ± 1.92 %
7.5 µM	7.62 ± 0.55 µM	101.56 ± 7.31 %
8.75 µM	9.38 ± 0.14 µM	107.23 ± 1.61 %
10 µM	10.77 ± 0.20 µM	107.74 ± 1.97 %
		⌀ 93.23 ± 2.99%

Tab. S5. Hemoglobin recovery with the Harboe method (100 µl sample volume) applying the corresponding equation to three dilution series. Recoveries with deviations of <15 % from 100 % recovery are highlighted in green. Recoveries with deviations of >15 % from 100 % recovery are marked in red.

Input concentration	Mean output concentration	Mean recovery rate
**Hemoglobin, 100 µl, microtiter plate, PBS**		
1.3 µM	0.87 ± 0.02 µM	68.88 ± 1.56 %
2.5 µM	1.89 ± 0.17 µM	74.51 ± 6.75 %
3.8 µM	3.04 ± 0.46 µM	79.91 ± 12.07 %
5.1 µM	4.33 ± 0.33 µM	85.50 ± 6.53 %
6.3 µM	5.13 ± 0.60 µM	80.95 ± 9.41 %
7.6 µM	7.00 ± 0.51 µM	92.07 ± 6.74 %
8.9 µM	8.44 ± 0.63 µM	95.14 ± 7.09 %
10.1 µM	9.77 ± 0.91 µM	96.46 ± 8.94 %
11.4 µM	10.98 ± 1.02 µM	96.27 ± 8.98 %
12.7 µM	12.26 ± 0.91 µM	96.77 ± 7.22 %
13.9 µM	13.89 ± 0.89 µM	99.69 ± 6.37 %
15.2 µM	14.93 ± 1.10 µM	98.23 ± 7.24 %
16.5 µM	16.40 ± 1.01 µM	99.60 ± 6.13 %
		⌀ 89.54 ± 7.31%
**Hemoglobin, 100 µl, microtiter plate, PBS + Na_2CO_3**		
2.5 µM	1.67 ± 0.64 µM	66.79 ± 25.58 %
3.75 µM	2.80 ± 0.55 µM	74.56 ± 14.65 %
5 µM	3.52 ± 0.64 µM	70.41 ± 12.78 %
6.25 µM	5.76 ± 0.14 µM	92.09 ± 2.19 %
7.5 µM	6.72 ± 0.22 µM	89.63 ± 2.97 %
8.75 µM	7.98 ± 0.65 µM	91.22 ± 7.40 %
10 µM	8.94 ± 0.32 µM	89.44 ± 3.18 %
11.25 µM	10.00 ± 0.07 µM	88.89 ± 0.63 %
12.5 µM	11.01 ± 0.12 µM	88.10 ± 0.99 %
13.75 µM	12.24 ± 0.60 µM	89.01 ± 4.33 %
15 µM	13.33 ± 0.68 µM	88.86 ± 4.50 %
16.25 µM	14.76 ± 0.55 µM	90.86 ± 3.38 %
17.5 µM	15.73 ± 1.90 µM	89.88 ± 10.88 %
		⌀ 85.36 ± 7.12 %
**Hemoglobin, 100 µl, microtiter plate, water + Na_2CO_3**		
2.5 µM	0.26 ± 0.04 µM	58.17 ± 4.73 %

3.75 µM	1.34 ± 0.09 µM	55.07 ± 4.42 %
5 µM	2.83 ± 0.12 µM	46.78 ± 6.81 %
6.25 µM	3.94 ± 0.44 µM	58.35 ± 2.65 %
7.5 µM	5.59 ± 0.40 µM	89.63 ± 2.97 %
8.75 µM	5.72 ± 0.11 µM	67.78 ± 2.34 %
10 µM	6.49 ± 0.16 µM	65.37 ± 1.23 %
11.25 µM	7.10 ± 0.44 µM	64.91 ± 1.61 %
12.5 µM	7.96 ± 0.20 µM	63.15 ± 3.88 %
13.75 µM	8.89 ± 0.83 µM	63.69 ± 1.57 %
15 µM	10.00 ± 0.27 µM	64.62 ± 6.00 %
16.25 µM	10.92 ± 0.82 µM	66.69 ± 1.78 %
17.5 µM	11.71 ± 0.71 µM	66.92 ± 4.06 %
		⌀ 62.21 ± 3.55 %
**Hemoglobin, 100 µl, microtiter plate, Tris-HCl**		
2.5 µM	2.83 ± 0.12 µM	113.03 ± 4.74 %
3.75 µM	3.94 ± 0.44 µM	105.09 ± 11.61 %
5 µM	5.59 ± 0.40 µM	111.84 ± 7.92 %
6.25 µM	6.97 ± 0.24 µM	111.49 ± 3.81 %
7.5 µM	8.67 ± 0.20 µM	115.58 ± 2.69 %
8.75 µM	10.21 ± 0.62 µM	116.69 ± 7.05 %
10 µM	11.78 ± 0.73 µM	117.83 ± 7.30 %
11.25 µM	13.18 ± 1.03 µM	117.14 ± 9.17 %
12.5 µM	14.62 ± 1.42 µM	116.97 ± 11.38 %
13.75 µM	16.35 ± 0.86 µM	118.94 ± 6.29 %
15 µM	17.45 ± 1.28 µM	116.35 ± 8.52 %
16.25 µM	19.02 ± 1.39 µM	117.06 ± 8.55 %
17.5 µM	20.58 ± 1.25 µM	117.57 ± 7.12 %
		⌀ 115.05 ± 2.63 %

Tab. S6. Heme and hemoglobin recovery using HPLC applying the respective equations derived from the calibration curves of heme (y = 2096511 x – 15660327) and hemoglobin (y = 34852476 x – 5618120) to three dilution series. Recoveries with deviations of <15 % from 100 % recovery are highlighted in green. Recoveries with deviations of >15 % from 100 % recovery are marked in red.

Input concentration	Mean output concentration	Mean recovery rate
**Heme determination with *y* = 2096511 *x* – 15660327**		
8 nmol	8.56 ± 0.18 nmol	107.03 ± 2.23 %
10 nmol	9.67 ± 0.25 nmol	96.68 ± 2.46 %
12 nmol	11.49 ± 0.33 nmol	95.78 ± 2.72 %
14 nmol	13.76 ± 0.93 nmol	98.30 ± 6.61 %
16 nmol	16.51 ± 1.53 nmol	103.21 ± 9.55 %
18 nmol	19.95 ± 3.40 nmol	110.82 ± 18.90 %
		⌀ 101.97 ± 7.08 %
**Hemoglobin determination with *y* = 35375434 *x* – 6495339**		
0.2 nmol	0.25 ± 0.02 nmol	124.97 ± 10.53 %
0.3 nmol	0.34 ± 0.05 nmol	112.19 ± 18.06 %
0.4 nmol	0.39 ± 0.04 nmol	96.74 ± 9.62 %
0.6 nmol	0.56 ± 0.03 nmol	93.99 ± 4.31 %
1.2 nmol	1.14 ± 0.11 nmol	94.76 ± 9.40 %
1.8 nmol	1.77 ± 0.14 nmol	98.12 ± 8.02 %
2.4 nmol	2.46 ± 0.21 nmol	102.47 ± 8.83 %
		⌀ 103.32 ± 9.83 %

Tab. S7. Heme recovery using ESI-MS applying the equation derived from the calibration curve of heme (y = 48564 x + 46751) to three dilution series.

Input concentration	Mean output concentration	Mean recovery rate
**Heme determination with *y* = 48564 *x* + 46751**		
0.8 pmol	0.24 ± 0.13 pmol	30.61 ± 16.31 %
1.6 pmol	1.34 ± 0.18 pmol	85.83 ± 11.61 %
3.2 pmol	3.19 ± 0.13 pmol	101.95 ± 4.30 %
6.3 pmol	6.84 ± 0.25 pmol	109.52 ± 4.01 %
12.5 pmol	12.97 ± 1.38 pmol	103.73 ± 11.01 %
25.0 pmol	24.64 ± 0.45 pmol	98.56 ± 1.79 %
		⌀ 88.37 ± 8.17 %

Tab. S8. HPLC-based heme recovery from hemoglobin samples using the equation derived from the calibration curve of heme ($y = 2096511\ x – 15660327$) to three series of hemoglobin samples.

Input concentration	Mean output concentration	Mean Hb:heme ratio
**Heme determination with *y* = 2096511 *x* – 15660327**		
1.2 nmol Hb	9.07 ± 0.35 nmol heme	1:7.56
1.8 nmol Hb	10.16 ± 0.53 nmol heme	1:5.64
2.4 nmol Hb	11.30 ± 0.80 nmol heme	1:4.71
		⌀ 1:5.97

Tab. S9. Heme and hemoglobin recovery with the pyridine hemochromogen assay applying the calculated extinction coefficient of 34.1 $mM^{-1}\ cm^{-1}$ (heme; 556 nm) and 80.4 $mM^{-1}\ cm^{-1}$ (hemoglobin; 556 nm) to three dilution series. A path length of 1 cm was considered. Recoveries with deviations of <15 % from 100 % recovery are highlighted in green. Recoveries with deviations of >15 % from 100 % recovery are marked in red.

Input concentration	Mean output concentration (calculated by ε_{556})	Mean recovery rate
**Heme at 556 nm, cuvette**		
7.5 µM	3.58 ± 1.30 µM	47.70 ± 17.27 %
12.5 µM	7.88 ± 1.78 µM	63.03 ± 14.22 %
17.5 µM	12.43 ± 2.50 µM	71.05 ± 14.27 %
25 µM	19.33 ± 2.70 µM	77.30 ± 10.81 %
30 µM	25.28 ± 2.92 µM	84.26 ± 9.74 %
37.5 µM	34.22 ± 3.61 µM	91.26 ± 9.64 %
		⌀ 72.43 ± 12.66 %
**Hemoglobin at 556 nm, cuvette**		
1.3 µM	1.25 ± 0.75 µM	95.99 ± 8.89 %
2.5 µM	2.59 ± 0.64 µM	103.48 ± 9.21 %
3.9 µM	3.71 ± 0.73 µM	95.04 ± 4.70 %
5.2 µM	4.99 ± 1.41 µM	95.99 ± 3.98 %
7.6 µM	7.55 ± 1.31 µM	99.34 ± 2.87 %
10.1 µM	9.87 ± 1.62 µM	97.74 ± 4.23 %
12.7 µM	12.77 ± 0.32 µM	100.51 ± 2.55 %
		⌀ 98.30 ± 5.20 %

Tab. S10. Heme and hemoglobin recovery with the pyridine hemochromogen assay applying the obtained linear regression equations for the absorbance at 556 nm (reduced sample) to three dilution series. Recoveries with deviations of <15 % from 100 % recovery are highlighted in green.

Input concentration	Mean output concentration	Mean recovery rate
**Heme determination with *y* = 0.034 *x* – 0.161**		
7.5 µM	8.32 ± 1.30 µM	110.98 ± 17.32 %
12.5 µM	12.64 ± 1.78 µM	101.10 ± 14.26 %
17.5 µM	17.21 ± 2.50 µM	98.32 ± 14.31 %
25 µM	24.12 ± 2.71 µM	96.47 ± 10.84 %
30 µM	30.09 ± 2.93 µM	100.29 ± 9.76 %
37.5 µM	39.06 ± 3.62 µM	104.16 ± 9.66 %
		⌀ 101.89 ± 12.69 %
**Hemoglobin determination with *y* = 0.080 *x* + 0.007**		
1.3 µM	1.16 ± 0.75 µM	89.30 ± 8.89 %
2.5 µM	2.50 ± 0.64 µM	100.00 ± 9.21 %
3.9 µM	3.62 ± 0.73 µM	92.81 ± 4.70 %
5.2 µM	4.90 ± 1.41 µM	94.32 ± 3.98 %
7.6 µM	7.46 ± 1.31 µM	98.19 ± 2.87 %
10.1 µM	9.78 ± 1.62 µM	96.88 ± 4.23 %
12.7 µM	12.68 ± 0.32 µM	95.90 ± 2.55 %
		⌀ 95.90 ± 5.20 %

Tab. S11. Heme and hemoglobin recovery with the pyridine hemochromogen assay applying the obtained linear regression equations for the absorbance difference between the reduced (at 556 nm) and oxidized (at 540 nm) sample to three dilution series. Recoveries with deviations of <15 % from 100 % recovery are highlighted in green.

Input concentration	Mean output concentration	Mean recovery rate
**Heme determination with *y* = 0.030 *x* – 0.141**		
7.5 µM	8.17 ± 1.22 µM	108.96 ± 16.26 %
12.5 µM	12.26 ± 1.67 µM	98.11 ± 13.34 %
17.5 µM	16.79 ± 2.39 µM	95.95 ± 13.64 %
25 µM	23.54 ± 2.59 µM	94.17 ± 10.36 %
30 µM	29.36 ± 2.75 µM	97.85 ± 9.18 %
37.5 µM	38.10 ± 3.48 µM	101.59 ± 9.27 %
		⌀ 99.44 ± 12.01 %
**Hemoglobin determination with *y* = 0.068 *x* – 0.009**		
1.3 µM	1.35 ± 0.12 µM	103.88 ± 9.32 %
2.5 µM	2.66 ± 0.22 µM	106.51 ± 8.63 %
3.9 µM	3.78 ± 0.19 µM	96.99 ± 4.95 %
5.2 µM	5.08 ± 0.19 µM	97.69 ± 3.70 %
7.6 µM	7.63 ± 0.21 µM	100.40 ± 2.73 %
10.1 µM	9.92 ± 0.45 µM	98.26 ± 4.48 %
12.7 µM	12.87 ± 0.33 µM	101.33 ± 2.56 %
		⌀ 100.72 ± 5.20 %

Tab. S12. Heme and hemoglobin recovery with the pyridine hemochromogen assay applying the for 557 nm in 1953 published extinction coefficient [7] of 34.7 mM^{-1} cm^{-1} (heme) to three dilution series. A path length of 1 cm was considered. Recoveries with deviations of <15 % from 100 % recovery are highlighted in green. Recoveries with deviations of >15 % from 100 % recovery are marked in red.

Input concentration	Mean output concentration (calculated by ε_{557})	Mean recovery rate
**Heme at 557 nm, cuvette**		
7.5 µM	3.44 ± 1.26 µM	45.85 ± 16.75 %
12.5 µM	7.59 ± 1.71 µM	60.71 ± 13.72 %
17.5 µM	11.97 ± 2.40 µM	68.40 ± 13.70 %
25 µM	18.62 ± 2.60 µM	74.47 ± 10.39 %
30 µM	24.32 ± 2.78 µM	81.08 ± 9.28 %
37.5 µM	32.92 ± 3.48 µM	87.79 ± 9.28 %
		⌀ 69.72 ± 12.19 %

Tab. S13. Heme and hemoglobin recovery with the pyridine hemochromogen assay applying the for $A_{557\ (red)}$ – $A_{540\ (ox)}$ published extinction coefficient [9] of 23.98 mM^{-1} cm^{-1} (heme) to three dilution series. A path length of 1 cm was considered. Recoveries with deviations of <15 % from 100 % recovery are highlighted in green. Recoveries with deviations of >15 % from 100 % recovery are marked in red.

Input concentration	Mean output concentration (calculated by $\varepsilon_{557-540}$)	Mean recovery rate
**Heme at D $A_{557\ (red)}$ – $A_{540\ (ox)}$, cuvette**		
7.5 µM	4.18 ± 4.18 µM	55.79 ± 19.90 %
12.5 µM	9.16 ± 2.03 µM	73.28 ± 16.21 %
17.5 µM	14.65 ± 2.89 µM	83.72 ± 16.50 %
25 µM	22.87 ± 3.14 µM	91.47 ± 12.56 %
30 µM	29.89 ± 3.30 µM	99.62 ± 11.00 %
37.5 µM	40.48 ± 4.22 µM	107.94 ± 11.26 %
		⌀ 85.30 ± 14.57 %

Tab. S14. Heme and hemoglobin recovery using the apoHRP-based assay with TMB applying the equations from the standard curves for DA/min at 652 nm to three dilution series. Recoveries with deviations of <15 % from 100 % recovery are highlighted in green.

Input concentration	Mean output concentration	Mean recovery rate
**Heme determination with *y* = 0.018 *x* – 0.538**		
33.33 nM	34.17 ± 2.01 nM	102.51 ± 6.03 %
35.71 nM	35.16 ± 0.30 nM	98.44 ± 0.83 %
38.10 nM	37.92 ± 0.96 nM	99.53 ± 2.52 %
40.48 nM	41.45 ± 0.62 nM	102.40 ± 1.54 %
42.86 nM	43.01 ± 2.31 nM	100.35 ± 5.38 %
		⌀ 100.65 ± 3.26 %
**Hemoglobin determination with *y* = 0.065 *x* – 0.145**		
2.86 nM	2.84 ± 0.31 nM	99.38 ± 10.80 %
3.33 nM	3.47 ± 0.40 nM	103.98 ± 11.91 %
3.81 nM	3.57 ± 0.34 nM	93.71 ± 9.01 %
4.17 nM	4.49 ± 0.30 nM	107.81 ± 7.10 %
4.52 nM	4.32 ± 0.54 nM	95.41 ± 11.94 %
4.76 nM	5.04 ± 0.28 nM	105.80 ± 5.78 %
5.24 nM	5.16 ± 0.34 nM	98.56 ± 6.43 %
		⌀ 100.66 ± 8.99 %

Tab. S15. Heme and hemoglobin recovery using the apoHRP-based assay with *o*-dianisidine hydrochloride applying the equations from the standard curves for DA/min at 457 nm to three dilution series. Recoveries with deviations of <15 % from 100 % recovery are highlighted in green. Recoveries with deviations of >15 % from 100 % recovery are marked in red.

Input concentration	Mean output concentration	Mean recovery rate
**Heme determination with $y = 0.002\,x – 0.025$**		
23.81 nM	19.41 ± 1.91 nM	81.55 ± 8.04 %
26.19 nM	24.07 ± 5.37 nM	91.88 ± 20.49 %
28.57 nM	24.30 ± 3.59 nM	85.03 ± 12.58 %
32.14 nM	24.74 ± 1.63 nM	76.98 ± 5.08 %
35.71 nM	29.59 ± 4.07 nM	82.85 ± 11.39 %
41.67 nM	36.22 ± 3.50 nM	86.92 ± 8.39 %
		⌀ 84.20 ± 11.00 %
**Hemoglobin determination with $y = 0.003\,x – 0.009$**		
9.52 nM	7.62 ± 1.67 nM	80.09 ± 17.58 %
13.1 nM	11.65 ± 2.42 nM	88.96 ± 18.46 %
17.86 nM	16.29 ± 2.74 nM	91.20 ± 15.32 %
26.19 nM	23.71 ± 0.47 nM	90.53 ± 1.81 %
28.57 nM	26.62 ± 1.51 nM	93.18 ± 5.30 %
32.14 nM	28.19 ± 1.82 nM	87.70 ± 5.67 %
35.71 nM	30.57 ± 2.03 nM	85.62 ± 5.69 %
		⌀ 88.18 ± 9.98 %

Tab. S16. Heme and hemoglobin recovery using the Hemin Assay Kit® applying the equations from the standard curves for ΔA/min at 570 nm to two dilution series. Recoveries with deviations of <15 % from 100 % recovery are highlighted in green. Recoveries with deviations of >15 % from 100 % recovery are marked in red.

Input concentration	Mean output concentration	Mean recovery rate
**Heme determination with *y* = 0.0004 *x* + 0.0001**		
8 nM	10.93 ± 7.97 nM	136.64 ± 81.39 %
16 nM	14.98 ± 5.21 nM	93.60 ± 24.80 %
24 nM	21.36 ± 10.62 nM	89.01 ± 36.13 %
32 nM	33.04 ± 14.76 nM	103.25 ± 37.13 %
40 nM	42.77 ± 33.78 nM	106.92 ± 68.96 %
		⌀ 105.88 ± 49.79 %
**Hemoglobin determination with *y* = 0.017 *x* – 0.018**		
1 nM	1.45 ± 0.34 nM	145.46 ± 34.17 %
2 nM	2.48 ± 0.38 nM	124.32 ± 19.04 %
4 nM	3.09 ± 1.54 nM	77.14 ± 38.51 %
6 nM	5.57 ± 0.42 nM	92.60 ± 7.01 %
8 nM	7.59 ± 5.11 nM	94.82 ± 63.82 %
10 nM	10.80 ± 1.24 nM	107.96 ± 12.37 %
		⌀ 107.05 ± 29.15 %

Tab. S17. Heme and hemoglobin recovery with the modified SLS method applying the obtained equations from the standard curves for the absorbance at 395 nm (heme) and 413 nm (hemoglobin), respectively, to three dilution series. Recoveries with deviations of <15 % from 100 % recovery are highlighted in green. Recoveries with deviations of >15 % from 100 % recovery are marked in red.

Input concentration	Mean output concentration	Mean recovery rate
**Heme determination with *y* = 0.027 *x* – 0.230**		
10 µM	11.74 ± 1.98 µM	117.43 ± 19.82 %
15 µM	14.42 ± 1.64 µM	96.12 ± 10.94 %
20 µM	19.13 ± 2.35 µM	95.66 ± 11.77 %
25 µM	23.42 ± 2.45 µM	93.67 ± 9.80 %
30 µM	28.42 ± 4.32 µM	94.72 ± 14.41 %
35 µM	34.01 ± 4.80 µM	97.18 ± 13.72 %
40 µM	39.62 ± 6.89 µM	99.05 ± 17.22 %
45 µM	45.67 ± 8.17 µM	101.48 ± 18.15 %
		⌀ 99.41 ± 14.48 %
**Hemoglobin determination with *y* = 0.151 *x* – 0.026**		
0.5 µM	0.53 ± 0.07 µM	105.74 ± 14.78 %
1 µM	1.00 ± 0.12 µM	99.96 ± 11.83 %
2 µM	1.92 ± 0.19 µM	96.06 ± 9.72 %
3 µM	3.03 ± 0.27 µM	100.91 ± 9.10 %
4 µM	3.90 ± 0.40 µM	97.51 ± 9.93 %
5 µM	5.15 ± 0.47 µM	102.98 ± 9.37 %
6 µM	5.91 ± 0.51 µM	98.44 ± 8.45 %
		⌀ 100.23 ± 10.46 %

Tab. S18. Heme and hemoglobin recovery rates with the Heme Assay Kit® applying the obtained linear regression equations for the absorbance at 400 nm to three dilution series. Recoveries with deviations of <15 % from 100 % recovery are highlighted in green.

Input concentration	Mean output concentration	Mean recovery rate
**Heme determination with *y* = 0.0391 *x* – 0.1743**		
8 µM	8.07 ± 0.35 µM	101.79 ± 7.01 %
12 µM	11.52 ± 0.47 µM	99.39 ± 7.07 %
16 µM	15.87 ± 0.36 µM	100.30 ± 3.98 %
20 µM	20.23 ± 0.23 µM	100.82 ± 1.11 %
24 µM	24.60 ± 0.26 µM	101.17 ± 2.11 %
28 µM	28.52 ± 0.34 µM	100.54 ± 1.94 %
32 µM	31.15 ± 0.05 µM	99.18 ± 1.71 %
		⌀ 100.46 ± 3.56 %
**Hemoglobin determination with *y* = 0.1014 *x* + 0.0151**		
0.5 µM	0.38 ± 0.10 µM	90.58 ± 23.73 %
1 µM	0.95 ± 0.11 µM	97.93 ± 14.94 %
2 µM	2.08 ± 0.19 µM	100.58 ± 14.74 %
4 µM	3.95 ± 0.18 µM	99.08 ± 6.30 %
6 µM	6.06 ± 0.33 µM	100.50 ± 9.40 %
8 µM	8.22 ± 0.34 µM	102.58 ± 4.69 %
10 µM	9.43 ± 0.87 µM	94.73 ± 7.23 %
		⌀ 98.00 ± 11.58 %

Tab. S19. Labile heme recovery using the Heme Assay Kit® applying equation 1 to four series of hemoglobin-heme mixtures in buffer solution (PBS, pH 7.4). H, heme; Hb, hemoglobin. Recoveries with deviations of <15 % from 100 % recovery are highlighted in green. Recoveries with deviations of >15 % from 100 % recovery are marked in red.

Input concentration	Heme recovery	Heme recovery rate
**Labile heme determination with Eq. 1**		
5 µM H	6.24 ± 0.48 µM	124.83 ± 9.65 %
5 µM H + 2 µM Hb	5.24 ± 1.58 µM	104.82 ± 31.69 %
5 µM H + 5 µM Hb	4.44 ± 2.24 µM	88.78 ± 44.74 %
5 µM H + 7 µM Hb	5.57 ± 3.82 µM	111.47 ± 76.42 %
8 µM H	8.37 ± 0.95 µM	104.65 ± 11.83 %
8 µM H + 2 µM Hb	8.33 ± 0.96 µM	104.13 ± 11.94 %
8 µM H + 5 µM Hb	6.91 ± 0.92 µM	86.43 ± 11.47 %
8 µM H + 7 µM Hb	3.79 ± 4.76 µM	47.35 ± 59.48 %
10 µM H	10.67 ± 1.30 µM	106.73 ± 12.99 %
10 µM H + 2 µM Hb	10.70 ± 1.12 µM	107.03 ± 11.23 %
10 µM H + 5 µM Hb	6.65 ± 3.09 µM	66.49 ± 30.91 %
10 µM H + 7 µM Hb	7.59 ± 4.19 µM	75.86 ± 41.91 %
15 µM H	15.75 ± 2.19 µM	105.02 ± 14.62 %
15 µM H + 2 µM Hb	14.83 ± 1.85 µM	98.88 ± 12.35 %
15 µM H + 5 µM Hb	12.36 ± 1.89 µM	82.42 ± 12.58 %
15 µM H + 7 µM Hb	11.81 ± 2.88 µM	78.71 ± 19.20 %
		⌀ 93.35 ± 25.81%

Tab. S20. Labile heme recovery was calculated applying equation 2 in six human plasma samples (**H21-H23** non-hemolytic, **H24-H26** hemolytic). The concentration of hemoglobin was determined using the Harboe method. To each human plasma sample increasing concentration of heme was added (2, 5, 10, 15 µM) to evaluate the labile heme recovery rate in human plasma samples. H, heme; Hb, hemoglobin. Recoveries with deviations of <15 % from 100 % recovery are highlighted in green. Recoveries with deviations of >15 % from 100 % recovery are marked in red.

Input concentration	Labile heme recovery	Labile heme recovery rate
**Labile heme determination with Eq. 1**		
**H21** (43.05 ± 0.17 µM H)		
**H21**+ 2 µM H	43.97 ± 0.25 µM	97.59 ± 0.55 %
**H21**+ 5 µM H	45.65 ± 0.64 µM	94.99 ± 1.42 %
**H21**+ 10 µM H	48.23 ± 0.64 µM	90.91 ±1.42 %
**H21**+ 15 µM H	54.24 ± 3.41 µM	93.44 ± 7.58 %
**H22** (44.70 ± 0.40 µM H)		
**H22**+ 2 µM H	45.10 ± 0.20 µM	96.57 ± 0.43 %
**H22**+ 5 µM H	46.30 ± 0.53 µM	99.13 ± 1.13 %
**H22**+ 10 µM H	48.51 ± 0.01 µM	102.87 ± 0.01 %
**H22**+ 15 µM H	52.60 ± 1.02 µM	112.62 ± 2.18 %
**H23** (46.03 ± 1.78 µM H)		
**H23**+ 2 µM H	45.87 ± 1.56 µM	95.38 ± 3.24 %
**H23**+ 5 µM H	45.58 ± 1.57 µM	94.90 ± 3.26 %
**H23**+ 10 µM H	49.94 ± 1.52 µM	103.97 ± 3.16 %
**H23**+ 15 µM H	55.82 ± 0.63 µM	116.22 ± 1.32 %
**H24** (64.35 ± 1.52 µM H)		
**H24**+ 2 µM H	64.16 ± 0.54 µM	96.71 ± 0.81 %
**H24**+ 5 µM H	65.68 ± 0.18 µM	98.99 ± 0.27 %
**H24**+ 10 µM H	70.41 ± 1.94 µM	106.12 ± 2.93 %
**H24**+ 15 µM H	55.82 ± 0.63 µM	120.19 ± 6.46 %
**H25** (88.41 ± 1.76 µM H)		
**H25**+ 2 µM H	90.35 ± 0.76 µM	99.94 ± 0.84 %
**H25**+ 5 µM H	90.29 ± 2.85 µM	99.87 ± 3.15 %
**H25**+ 10 µM H	99.96 ± 2.50 µM	110.56 ± 2.77 %
**H25**+ 15 µM H	114.98 ± 0.66 µM	127.17 ± 0.73 %
**H26** (71.98 ± 2.13 µM H)		
**H26**+ 2 µM H	71.29 ± 1.75 µM	96.36 ± 2.36 %
**H26**+ 5 µM H	71.18 ± 1.21 µM	96.22 ± 1.64 %
**H26**+ 10 µM H	75.20 ± 2.35 µM	101.65 ± 3.17 %

**H26**+ 15 µM H	88.49 ± 0.54 µM	119.61 ± 0.72 %
		⌀ 103.04 ± 9.71%

References

1. M.T. Hopp MT, B.F. Schmalohr, T. Kühl, M.S. Detzel, A. Wißbrock, D. Imhof, Heme determination and quantification methods and their suitability for practical applications and everyday use, Anal Chem. 92 (2020) 9429-9440.
2. M.T. Hopp, D.C. Rathod, K.H. Winn, S. Ambast, D. Imhof, Novel insights into heme binding to hemoglobin, Biol Chem. 403 (2022) 1055-1066.
3. D.F. Swinehart, The Beer-Lambert law. J Chem Educ. 39 (1962) 333.
4. H. Atamna, M. Brahmbhatt, W. Atamna, G.A. Shanower, J.M. Dhahbi, ApoHRP-based assay to measure intracellular regulatory heme, Metallomics. 7 (2015) 309-321.
5. A. Wißbrock, T. Kühl, K. Silbermann, A.J. Becker, O. Ohlenschläger, D. Imhof, Synthesis and evaluation of amyloid β derived and amyloid β independent enhancers of the peroxidase-like activity of heme, J Med Chem. 60 (2017) 373-385.
6. B.O. Keller, J. Sui, A.B. Young, R.M. Whittal. Interferences and contaminants encountered in modern mass spectrometry, Anal Chim Acta. 627(1) (2008) 71–81.
7. K.G. Paul, H. Theorell, A. Åkeson, The molar light absorption of pyridine ferroprotoporphyrin (pyridine haemochromogen), Acta Chem Scand. 7 (1953) 1284-1287.
8. International Council for Harmonisation of Technical Requirements for Pharmaceuticals for Human Use. ICH Topic Q2 (R1) Validation of analytical procedures: Text and methodology. June 1995 [Online]. Available from: https://www.ema.europa.eu/en/documents/scientific-guideline/ich-q-2-r1-validation-analytical-procedures-text-methodology-step-5_en.pdf
9. E.A. Berry, B.L. Trumpower, Simultaneous determination of hemes a, b, and c from pyridine hemochrome spectra, 161(1) Anal Biochem. (1987) 1-15.
